# Genetic analysis of uterine adenosarcomas and phyllodes tumors of the breast

**DOI:** 10.1002/1878-0261.12049

**Published:** 2017-05-16

**Authors:** Felipe C. Geyer, Kathleen A. Burke, Salvatore Piscuoglio, Charlotte K. Y. Ng, Anastasios D. Papanastasiou, Caterina Marchiò, Pier Selenica, Marcia Edelweiss, Melissa P. Murray, Edi Brogi, Robert A. Soslow, Brian P. Rubin, Larry Norton, Jorge S. Reis‐Filho, Britta Weigelt

**Affiliations:** ^1^ Department of Pathology Memorial Sloan Kettering Cancer Center New York NY USA; ^2^ Department of Medical Sciences University of Turin Italy; ^3^ Department of Pathology Cleveland Clinic Cleveland OH USA; ^4^ Department of Medicine Memorial Sloan Kettering Cancer Center New York NY USA

**Keywords:** genotypic–phenotypic correlation, gynecology, next‐generation sequencing, pan‐cancer analysis, pathology

## Abstract

Uterine adenosarcomas and breast phyllodes tumors (PTs) are morphologically similar, being composed of stromal projections in a leaf‐like fashion lined by epithelial cells. Here, we investigated whether their histologic similarities would be mirrored at the genetic level. The previously reported repertoires of somatic genetic alterations found in 19 adenosarcomas and 22 PTs (six benign, six borderline, and 10 malignant) were compared. PTs significantly more frequently displayed mutations affecting *MED12*, the *TERT* gene promoter and *bona fide* cancer genes, whereas adenosarcomas harbored a higher rate of *MDM2*/*CDK4* and *TERT* gene amplifications. Pathway analyses based on the genes affected by somatic genetic alterations in these tumors indicated that Wnt signaling likely plays a role in the biology of adenosarcomas and benign/borderline PTs. In conclusion, despite the differences at the gene level, PTs and adenosarcomas share remarkable morphologic similarities and enrichment for somatic genetic alterations affecting Wnt pathway‐related genes.

**Abbreviations**
HPF: high‐power field
MSK‐IMPACT: Memorial Sloan Kettering‐Integrated Mutation Profiling of Actionable Cancer Targets
PT: phyllodes tumor

## 1 Introduction

Uterine adenosarcomas and phyllodes tumors (PTs) of the breast are fibroepithelial lesions with remarkable morphologic similarities (Tan *et al*., 2012; Wells *et al*., 2014). Both entities display a typical leaf‐like architecture where finger‐like projections composed of a neoplastic mesenchymal component with varying degrees of atypia, cellularity, and proliferation are lined by a nonclonally related epithelial component (Piscuoglio *et al*., 2016a,b; Tan *et al*., 2012, 2015; Wells *et al*., 2014). The prognosis of patients affected by both entities is indeed largely defined by the features of the mesenchymal component (Tan *et al*., 2012; Wells *et al*., 2014), which has been shown to be the clonal and neoplastic component in adenosarcomas and PTs (Piscuoglio *et al*., 2016a,b; Tan *et al*., 2015).

Uterine adenosarcomas are mostly indolent lesions, with low‐grade histologic features and low recurrence rates (Carroll *et al*., 2014; McCluggage, 2010). Approximately 25% of adenosarcomas, however, may display sarcomatous overgrowth, which is often associated with higher pathologic stage and more aggressive clinical behavior (Carroll *et al*., 2014; McCluggage, 2010). Genetically, adenosarcomas are heterogeneous (Howitt *et al*., 2015; Piscuoglio *et al*., 2016a). A consistent finding has been a rate of 26–28% of amplifications affecting *MDM2*/*CDK4* (Howitt *et al*., 2015; Piscuoglio *et al*., 2016a). Additional recurrent alterations, although not detected in all or at similar rates across different studies, include mutations affecting PI3K pathway‐related genes, *ATRX* and *TP53*, and amplifications of *TERT* and *MYBL1* (Howitt *et al*., 2015; Piscuoglio *et al*., 2016a).

Akin to adenosarcomas, the majority of PTs have a good outcome, albeit some can display metastatic behavior (Tan *et al*., 2012, 2016). PTs are classified as benign, borderline, or malignant based on the histologic features of their mesenchymal component (Tan *et al*., 2012, 2016). Recent studies have revealed the molecular underpinning of PTs (Cani *et al*., 2015; Gatalica *et al*., 2016; Liu *et al*., 2016; Piscuoglio *et al*., 2016b; Tan *et al*., 2015), which are characterized by *MED12* mutations affecting exon 2 in around 60%, as well as recurrent mutations affecting *RARA*,* FLNA*, and *SETD2* (Cani *et al*., 2015; Piscuoglio *et al*., 2016b; Tan *et al*., 2015). *MED12* mutations are currently perceived as a founder genetic event in PTs, but are significantly more prevalent in benign than in malignant tumors (Piscuoglio *et al*., 2016b; Yoon *et al*., 2016). In contrast, *TERT* genetic alterations, which occur in around 55% of all PTs and include *TERT* promoter hotspot mutations and rare *TERT* gene amplification, are more frequent in malignant tumors (Piscuoglio *et al*., 2016b). Moreover, genetic alterations affecting *bona fide* cancer genes, such as *TP53*,* RB1*, and *EGFR*, appear to be restricted to borderline and malignant PTs (Piscuoglio *et al*., 2016b; Tan *et al*., 2015). Current data therefore suggest that the genetic make‐up of PTs is strongly associated with histologic grade.

Genetic analyses of human neoplasms have demonstrated striking examples of genotypic–phenotypic correlations. For instance, regardless of the site of origin, recurrent *MYB*‐*NFIB* fusion genes (Martelotto *et al*., 2015; Persson *et al*., 2009) and *MAML2* rearrangements (O'Neill, 2009) underpin adenoid cystic and mucoepidermoid carcinomas, respectively. Moreover, tumors arising in distinct organs can converge into common genomic subtypes, such as lung squamous, head and neck, and a subset of bladder carcinomas, which have been shown to display numerous genetic and transcriptomic similarities in multiplatform pan‐cancer analyses (Hoadley *et al*., 2014). Likewise, gynecologic high‐grade serous carcinomas and basal‐like breast cancers share a similar genomic signature, with highly recurrent *TP53* mutations, frequent *BRCA1* inactivation, 5q losses, and 8q gains (Cancer Genome Atlas Research Network *et al*., 2013).

Given the histologic similarities of uterine adenosarcomas and PTs, we have posited that these tumors would display a similar repertoire of somatic mutations, providing another example of a genotypic–phenotypic correlation. Hence, using massively parallel sequencing data previously generated by our group (Piscuoglio *et al*., 2016a,b), we compared the repertoire of somatic genetic alterations in uterine adenosarcomas and PTs of the breast. Given that the equivalent of malignant PT differ from the vast majority of adenosarcomas, we also performed hypothesis‐generating analyses between adenosarcomas and PTs, with the latter being stratified according to histologic grade.

## 2 Materials and methods

### 2.1 Cases

The cases included in this study have been previously described elsewhere (Piscuoglio *et al*., 2016a,b). Here, we report on a re‐analysis of previously published massively parallel sequencing data reported by Piscuoglio *et al*. (2016a,b), including 19 uterine adenosarcomas (six with stromal overgrowth) (Piscuoglio *et al*., 2016a) and 22 PTs (six benign, six borderline, and 10 malignant, Table 1; Piscuoglio *et al*., 2016b). Three malignant PTs included in Piscuoglio *et al*. (2016b) were not included in this re‐analysis as they were analyzed by a sequencing assay targeting a smaller set of genes than the remaining samples. Details of pathology review and tissue microdissection are described in the original publications (Piscuoglio *et al*., 2016a,b). Briefly, representative sections of each PT and uterine adenosarcoma were microdissected using a sterile needle under a stereomicroscope to ensure a tumor cell content > 80%. Tissue sections of normal breast or lymph nodes from patients with PT and of normal myometrium from patients with adenosarcoma were also microdissected to ensure the lack of neoplastic cells in normal tissue samples. In the normal breast sections, we have preferentially retrieved DNA from areas rich in stromal cells, inflammatory infiltrate, and adipose tissue rather than breast terminal duct‐lobular units, ducts and lobules. PTs were classified as benign, borderline, or malignant according to the latest World Health Organization criteria (Tan *et al*., 2012). Given the lack of an internationally accepted criteria for the grading of uterine adenosarcomas, ‘low‐grade’ adenosarcomas were defined as those tumors composed of small mesenchymal cells lacking pleomorphism, with a mitotic index < 10 mitotic figures/10 high‐power fields (HPF); ‘intermediate‐grade’ adenosarcomas were classified as such if the tumor displayed pleomorphic mesenchymal cells and a mitotic index < 10 mitotic figures/10 HPF; and ‘high‐grade’ adenosarcomas were defined on the basis of the presence of pleomorphic mesenchymal cells and a mitotic index > 10 mitotic figures/10 HPF (Piscuoglio *et al*., 2016a).

**Table 1:** Clinicopathologic features of uterine adenosarcomas and phyllodes tumors of the breast included in this study

	Uterine adenosarcomas		Phyllodes tumors of the breast	
	*n* = 19	%	*n* = 22	%
Grade^a^				
Low/benign	13	68	6	27
Intermediate/borderline	4	21	6	27
High/malignant	2	11	10	46
Stromal overgrowth				
Absent	13	68	19	86
Present	6	32	3	14
Heterologous components				
Absent	16	84	21	95
Present	3	16	1	5

a Grading of phyllodes tumors of the breast was performed according to WHO criteria (Tan *et al*., 2012). Grading of uterine adenosarcomas was performed as described in Piscuoglio *et al*. (2016a).

### 2.2 Whole‐exome and targeted massively parallel sequencing

The massively parallel sequencing data were retrieved from SRA (accession numbers SRP063459 and SRP063461 for adenosarcomas and SRP062618 for PTs). Six adenosarcomas had been subjected to whole‐exome sequencing and 13 adenosarcomas and all PTs had been subjected to targeted massively parallel sequencing using the Memorial Sloan Kettering‐Integrated Mutation Profiling of Actionable Cancer Targets (MSK‐IMPACT) sequencing assay, targeting all coding regions and selected intronic and promoter regions of 341 (nine adenosarcomas) or 410 (three adenosarcomas and 22 PTs) key cancer genes. The 341‐gene panel was concurrently present in the 410‐gene panel and is considered for the re‐analysis here described (Table S1). Sequencing data were analyzed as previously described (Cibulskis *et al*., 2013; De Mattos‐Arruda *et al*., 2014; Koboldt *et al*., 2012; Piscuoglio *et al*., 2016a,b; Robinson *et al*., 2011; Saunders *et al*., 2012). The functional effect of each missense single nucleotide variant was investigated as previously described (Piscuoglio *et al*., 2016a,b), using a combination of three mutation function predictors, namely MutationTaster, CHASM, and FATHMM (Carter *et al*., 2009; Martelotto *et al*., 2014; Schwarz *et al*., 2010). Genes affected by nonpassenger mutations were assessed for their presence in three cancer gene datasets, included in Kandoth *et al*. (2013), the Cancer Gene Census (Futreal *et al*., 2004) and Lawrence *et al*. (2014). Hotspot single nucleotide variants were annotated according to the hotspot list from Chang *et al*. (2016). Allele‐specific copy number alterations were identified using FACETS as previously described (Geyer *et al*., 2016; Shen and Seshan, 2016). ABSOLUTE (v1.0.6) (Carter *et al*., 2012) was employed to define the cancer cell fraction of each mutation, based on the number of reads supporting the reference and the alternate alleles and the segmented Log_2_ ratio from targeted capture massively parallel sequencing as input as previously described (Geyer *et al*., 2016). Solutions from ABSOLUTE were manually reviewed as recommended (Carter *et al*., 2012; Landau *et al*., 2013). A mutation was classified as clonal if its probability of being clonal was > 50% (Landau *et al*., 2013) or if the lower bound of the 95% confidence interval of its cancer cell fraction was > 90% (Geyer *et al*., 2016). Mutations that did not meet the above criteria were considered subclonal.

### 2.3 Statistical analysis and comparisons

Comparisons of overall mutation and copy number alteration rates were made using Mann–Whitney *U*‐test. Comparisons of the frequencies of alterations affecting specific genes were made using Fisher's exact tests. Depth of coverage was compared using Student's *t*‐test. All tests were two‐tailed. A confidence interval of 95% was adopted for all tests.

### 2.4 Pathway analysis

To investigate whether genetic alterations detected in uterine adenosarcomas and PTs of the breast would result in the activation of similar molecular pathways, a pathway analysis was performed using a combination of gProfiler (Reimand *et al*., 2016), MsigDB (Subramanian *et al*., 2005), and DAVID (Huang da *et al*., 2009a,b). Genes affected by nonsynonymous somatic mutations, amplifications, and/or homozygous deletions were input as lists for adenosarcomas and PTs, and the latter stratified by grade as well, and the top ten significantly altered pathways were reported. In gProfiler and DAVID (Huang da *et al*., 2009a,b), we employed the list of 341 genes sequenced in all samples in this study (Table S1) as the background gene list. For compensation of this background in MsigDB (Subramanian *et al*., 2005), the list of 341 genes was analyzed and any significantly altered pathway detected were removed from the list of significantly altered pathways of the other gene sets. The remaining pathways were compared across groups.

## 3 Results

### 3.1 Cases

**Figure 1:**
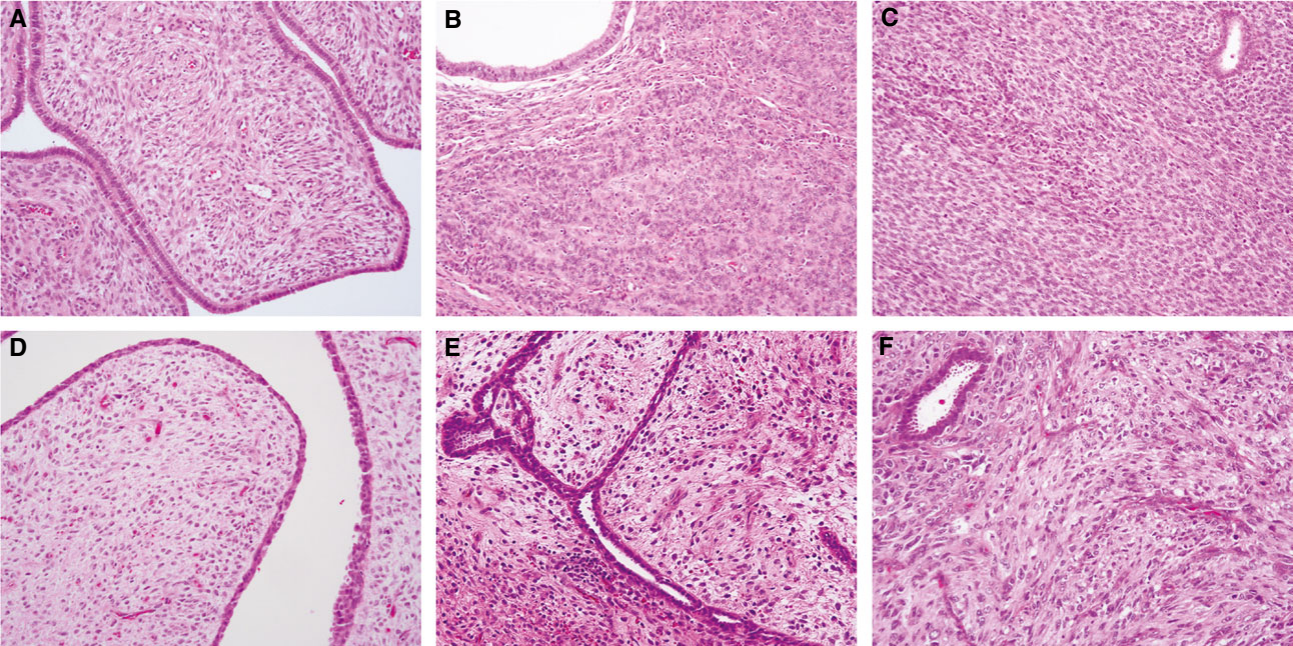
Representative micrographs of uterine adenosarcomas and phyllodes tumors of the breast. (A) Low‐grade adenosarcoma. (B) Intermediate‐grade adenosarcoma with sex cord‐like features (C) High‐grade adenosarcoma with stromal overgrowth. (D) Benign phyllodes tumor. (E) Borderline phyllodes tumor. (F) Malignant phyllodes tumor with stromal overgrowth. Magnification, 100 ×.

The pathologic features of uterine adenosarcomas and PTs of the breast have been previously described (Piscuoglio *et al*., 2016a,b) and are summarized in Table 1 and illustrated in Fig. 1. Briefly, benign PTs (*n* = 6) displayed stromal components with mild atypia, mild‐to‐moderate cellularity, and mitotic rates ranging from 0 to 2 (median 0.5) mitoses/10 HPF. Borderline PTs (*n* = 6) displayed mostly moderate stromal cellularity, varying levels of stromal atypia, and mitotic rates ranging from 2 to 7 (median 5.5) mitoses/10 HPF. The majority of malignant PTs (*n* = 10) displayed marked stromal cellularity, moderate to marked stromal atypia, and mitotic rates of >10 mitoses/10 HPF. A stromal heterologous component was present in one malignant PT, in the form of liposarcoma (MaPT06). By contrast, all but two uterine adenosarcomas were of low (*n* = 13) or intermediate grade (*n* = 4), with mitotic rate lower than 10 mitoses/10 HPF. Stromal overgrowth was present in six cases, mostly in intermediate‐ or high‐grade adenosarcomas. Stromal heterologous components were present in three cases; two cases displayed rhabdomyoblastic differentiation (AS3 and BAS16), whereas one case displayed osseous differentiation (AS6).

### 3.2 Uterine adenosarcomas harbor lower mutation burden than PTs of the breast

**Figure 2:**
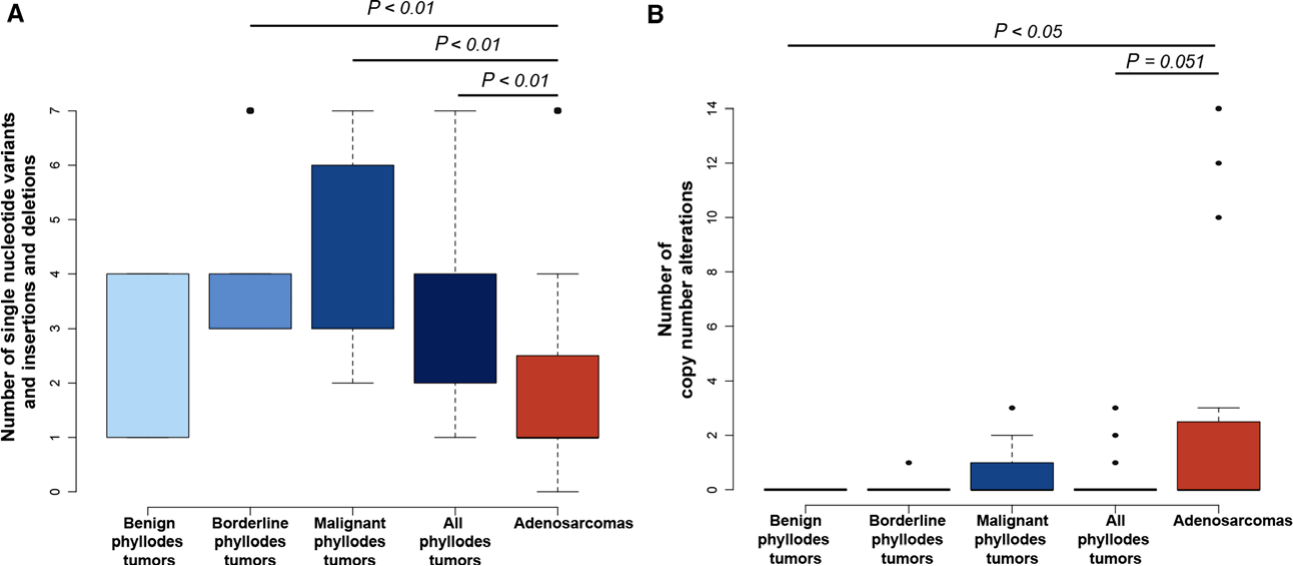
Number of single nucleotide variants and insertions and deletions, and of copy number alterations in uterine adenosarcomas and phyllodes tumors of the breast. (A) Box‐and‐whisker plots depicting the number of single nucleotide variants and insertions and deletions in uterine adenosarcomas, all phyllodes tumors, benign phyllodes tumors, borderline phyllodes tumors, and malignant phyllodes tumors. (B) Box‐and‐whisker plots depicting the number of copy number alterations in uterine adenosarcomas, all phyllodes tumors, benign phyllodes tumors, borderline phyllodes tumors, and malignant phyllodes tumors. *P* values based on Mann–Whitney *U*‐test.

Whole‐exome sequencing of six adenosarcomas yielded a median coverage of 335x (range 277x–403x). MSK‐IMPACT yielded comparable coverage between 13 adenosarcomas (median 649x, range 163x–1624x) and 22 PTs (median 581, range 308x–1114x). The coverage was not significantly different across the different sequencing methods (Student's *t*‐test, *P* > 0.05). The overall rate of nonsynonymous somatic mutations affecting the 341 genes present in all platforms differed significantly between adenosarcomas (median 1, range 1–6) and all PTs (median: 3, range 1–7; Mann–Whitney *U*‐test, *P* = 0.0012; Fig. 2A, Table S2). Given that the mutation rate in PTs increases according to grade (Piscuoglio *et al*., 2016b), we made a comparison between the mutation rates in adenosarcomas and PTs stratified according to histologic grade. The overall nonsynonymous somatic mutation rate in adenosarcomas was comparable to that of benign PTs (median 1, range 1–3; Mann–Whitney *U*‐test, *P* > 0.05, Fig. 2A), but significantly lower than that detected in borderline (median 4, range 3–7; Mann–Whitney *U*‐test, *P* = 0.0056) and malignant PTs (median 3.5, range 2–7; Mann–Whitney *U*‐test, *P* = 0.0014; Fig. 2A). At the copy number level, however, adenosarcomas displayed a trend for a higher number of amplifications and homozygous deletions than all PTs (Mann–Whitney *U*‐test, *P* = 0.051; Figs 2B and S1). When PTs were stratified by grade, adenosarcomas displayed significantly higher numbers of gene copy number alterations than benign PTs (Mann–Whitney *U*‐test, *P* = 0.0495; Fig. 2B); however, there was no statistically significant difference when adenosarcomas were compared to borderline and malignant PTs, given that, akin to the mutation burden, the number of copy number alterations in PTs is also significantly associated with grade (Piscuoglio *et al*., 2016b).

### 3.3 Uterine adenosarcomas and PTs of the breast harbor distinct repertoires of somatic genetic alterations

**Figure 3:**
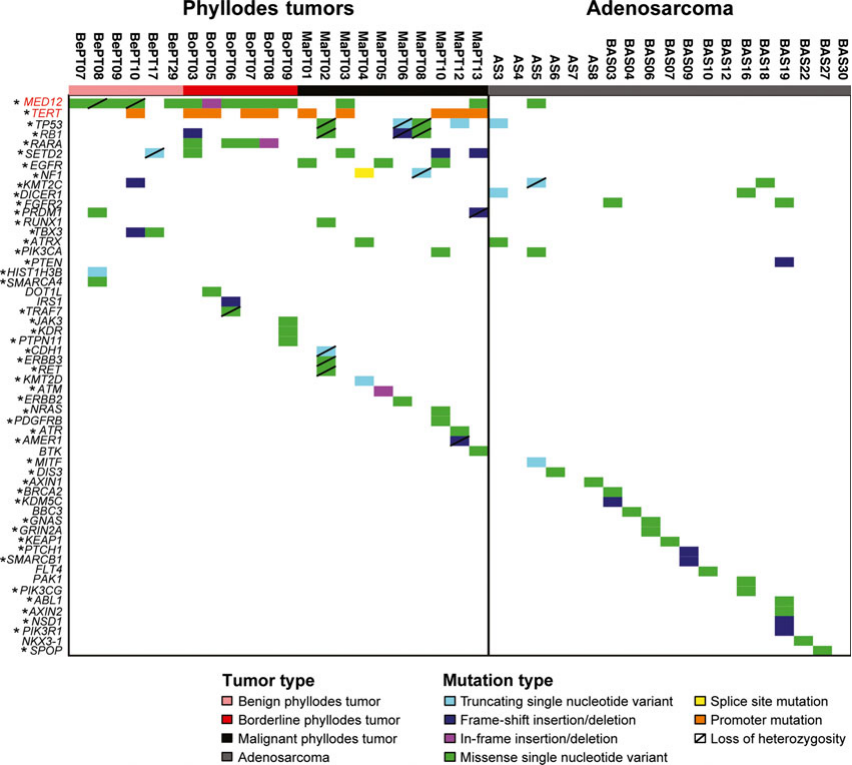
Nonsynonymous somatic mutations detected by massively parallel sequencing in uterine adenosarcomas and phyllodes tumors of the breast. Heatmap indicating the somatic mutations identified in the uterine adenosarcomas (*n *= 19) and phyllodes tumors of the breast (*n *= 22). Each column represents one sample; mutated genes are reported in rows. Mutation types are color‐coded according to the legend. Loss of heterozygosity of the wild‐type allele of a mutated gene is represented by a diagonal bar. Genes in red denote significant differences between adenosarcomas and phyllodes tumors (*P* < 0.05, Fisher's exact tests). Cancer genes included in Kandoth *et al*. (2013), the Cancer Gene Census (Futreal *et al*., 2004), and Lawrence *et al*. (2014) are highlighted by an asterisk preceding the gene name.

Phyllodes tumors of the breast, as a group, displayed a significantly higher frequency of mutations affecting *MED12* (59% vs 5%, Fisher's exact test, *P* = 0.0002) and the promoter of *TERT* (45% vs 0, Fisher's exact test, *P* = 0.0006, Fig. 3, Table S2) than adenosarcomas. Notably, one adenosarcoma displayed a *MED12* mutation, but the latter was not in exon 2, the exon recurrently affected in PTs (Cani *et al*., 2015; Piscuoglio *et al*., 2016b; Tan *et al*., 2015). *STED2* and *RARA*, which are known to be recurrently mutated in PTs and are potentially related to their development, were not mutated in adenosarcomas, but these differences did not reach statistical significance (23% vs 0, *P* = 0.0506; 18% vs 0, *P* = 0.1105, respectively, Fisher's exact tests). Mutations affecting additional *bona fide* cancer genes were numerically more frequent in PTs than in adenosarcomas, such as *TP53* (18% vs 5%), *RB1* (18% vs 0), and *EGFR* (13% vs 0), although again these differences were not statistically significant (Fisher's exact tests, *P* > 0.05). Several cancer genes rarely mutated in adenosarcomas, such as *DICER1* (11%), *FGFR2* (11%), and *BRCA2* (5%), were not mutated in any of the PTs analyzed.

### 3.4 The repertoire of somatic mutations affecting uterine adenosarcomas and PTs of the breast stratified by grade

As an exploratory hypothesis‐generating analysis, we compared the frequencies of alterations affecting individual genes in adenosarcomas and PTs stratified by grade. Given that benign PTs display a significantly lower mutation burden than malignant PTs (Piscuoglio *et al*., 2016b) and lack mutations affecting *bona fide* cancer genes (Piscuoglio *et al*., 2016b; Tan *et al*., 2015), only *MED12* remained significantly more frequently mutated in benign PTs than in adenosarcomas (83% vs 5%; Fisher's exact test, *P* = 0.0006; Fig. S2A). In contrast, as *MED12* exon 2 mutations are inversely correlated with the grade of PTs (Piscuoglio *et al*., 2016b) and mutations affecting the *TERT* gene promoter (‐124C>T) and *bona fide* cancer genes are more prevalent in malignant PTs (Piscuoglio *et al*., 2016b), a comparison between malignant PTs and adenosarcomas revealed that *TERT* promoter (50% vs 0, Fisher's exact test, *P* = 0.0021), *RB1* (30% vs 0, Fisher's exact test, *P* = 0.0328), *TP53* (40% vs 5%, Fisher's exact test, *P* = 0.0357), and *EGFR* (30% vs 0, Fisher's exact test, *P* = 0.0328, Fig. S2B) mutations were significantly more frequent in the former. As expected, a comparison between borderline PTs and adenosarcomas revealed intermediate results, with mutations affecting *MED12* (100% vs 5%, Fisher's exact test, *P* = 3.952 × 10^−5^), *TERT* promoter (67% vs 0, Fisher's exact test, *P* = 0.0012), and *RARA* (67% vs 0, Fisher's exact test, *P* = 0.0012, Fig. S2C) being significantly more frequent in borderline PTs than in adenosarcomas.

As adenosarcomas are mostly low‐grade neoplasms (Table 1) and morphologically more similar to benign and borderline rather than malignant PTs, we also performed an exploratory, hypothesis‐generating comparison of the mutation repertoire of benign and borderline PTs as a group (*n* = 12) with that of adenosarcomas. *MED12* (92% vs 5%, Fisher's exact test, *P* = 1.620 × 10^−6^), *TERT* (42% vs 0, Fisher's exact test, *P* = 0.0047), and *RARA* (33% vs 0, Fisher's exact test, *P* = 0.0157, Fig. S2D) were significantly more frequently mutated in that subset of PTs than in adenosarcomas, whereas no gene was significantly more frequently mutated in adenosarcomas.

As a third exploratory hypothesis‐generating analysis, we compared all PTs *versus* adenosarcomas stratified by grade (13 low‐grade and six intermediate‐/high‐grade adenosarcomas pooled together), as well as benign PTs *versus* low‐grade adenosarcomas, and malignant PTs *versus* intermediate‐/high‐grade adenosarcomas. These comparisons revealed similar findings, with significant differences being restricted to mutations affecting *MED12* and *TERT* (*P* < 0.05, Fisher's exact tests, Fig. S4).

### 3.5 Uterine adenosarcomas and PTs of the breast differ in their pattern of focal amplifications

**Figure 4:**
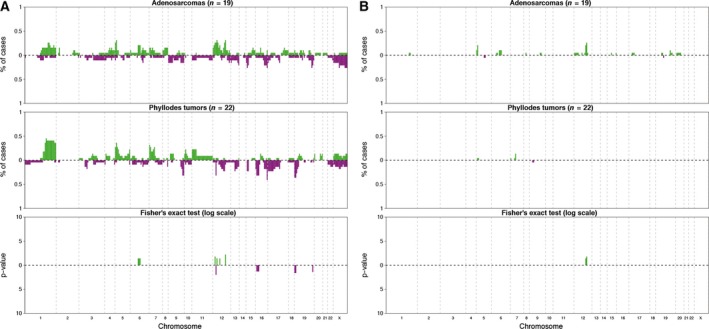
Comparisons of the frequency of copy number alterations identified in uterine adenosarcomas and phyllodes tumors of the breast. (A) The frequency plots highlight the presence of recurrent gains and losses in uterine adenosarcomas (top) and phyllodes tumors (middle). Significant differences (Fisher's exact test *P* < 0.05) are plotted in the bottom panel. On the *y*‐axis, the proportion of samples in which gains (green bars) or losses (purple bars) were identified is plotted according to genomic location (*x*‐axis). (B) The frequency plots highlight the presence of recurrent amplifications in uterine adenosarcomas (top) and phyllodes tumors (middle). Significant differences (Fisher's exact test *P* < 0.05) are plotted in the bottom panel. The proportion of samples (*y*‐axis) in which gains (green bars) or losses (purple bars) were identified is plotted according to genomic location (*x*‐axis).

A genome‐wide copy number alteration analysis revealed a rather similar pattern of low‐level gains and losses between adenosarcomas and all PTs (Fig. 4A). Focal regions on 6q, 12p, and 12q were significantly more frequently gained in adenosarcomas, whereas focal regions on 12q, 16p, and 19p were significantly more frequently lost in PTs (Fisher's exact tests, *P* < 0.05; Fig. 4A). Focal amplifications in 12q14.1‐15, encompassing the loci of *MDM2* and *CDK4*, were significantly more prevalent in adenosarcomas than in PTs (26% vs 0, Fisher's exact test, *P* = 0.0155; Fig. 4B). Moreover, amplifications encompassing the *TERT* gene locus on 5p15.33 were more frequent in adenosarcomas than in PTs; however, this difference did not reach statistical significance (21% vs 5%; Fisher's exact test, *P* = 0.1644).

When PTs were stratified according to grade, no regions were significantly differentially altered between benign PTs and adenosarcomas (Fisher's exact tests, *P* > 0.05; Fig. S3A,B), whereas a focal region on 7p11.2 including *EGFR* was significantly more frequently amplified in malignant PTs than in adenosarcomas (30% vs 0, Fisher's exact tests, *P* = 0.0328, Fig. S3C,D). Borderline PTs displayed a significantly higher frequency of 1q gains and focal 12p losses than adenosarcomas (Fisher's exact tests, *P* < 0.05), but no region was significantly differentially amplified (Fisher's exact tests, *P* > 0.05, Fig. S3E,F).

When benign and borderline PTs were combined together as a group, a focal region on 12p was significantly more frequently lost in this subset of PTs than in adenosarcomas (Fisher's exact tests, *P* < 0.05, Fig. S3G). Significant differences were not found between these two groups in terms of homozygous deletions and amplifications, although *MDM2*/*CDK4* amplifications were numerically more frequent in adenosarcomas (32% vs 0, Fisher's exact tests, *P* = 0.058, Fig. S3H).

As a final exploratory hypothesis‐generating analysis, we repeated the copy number analysis stratifying adenosarcomas by grade. Amplifications of *MDM2*/*CDK4* remained significantly more prevalent in low‐grade adenosarcomas than in all PTs (Fisher's exact test, *P* = 0.0242, Fig. S5).

### 3.6 Pathway analysis in uterine adenosarcomas and PTs of the breast

**Figure 5:**
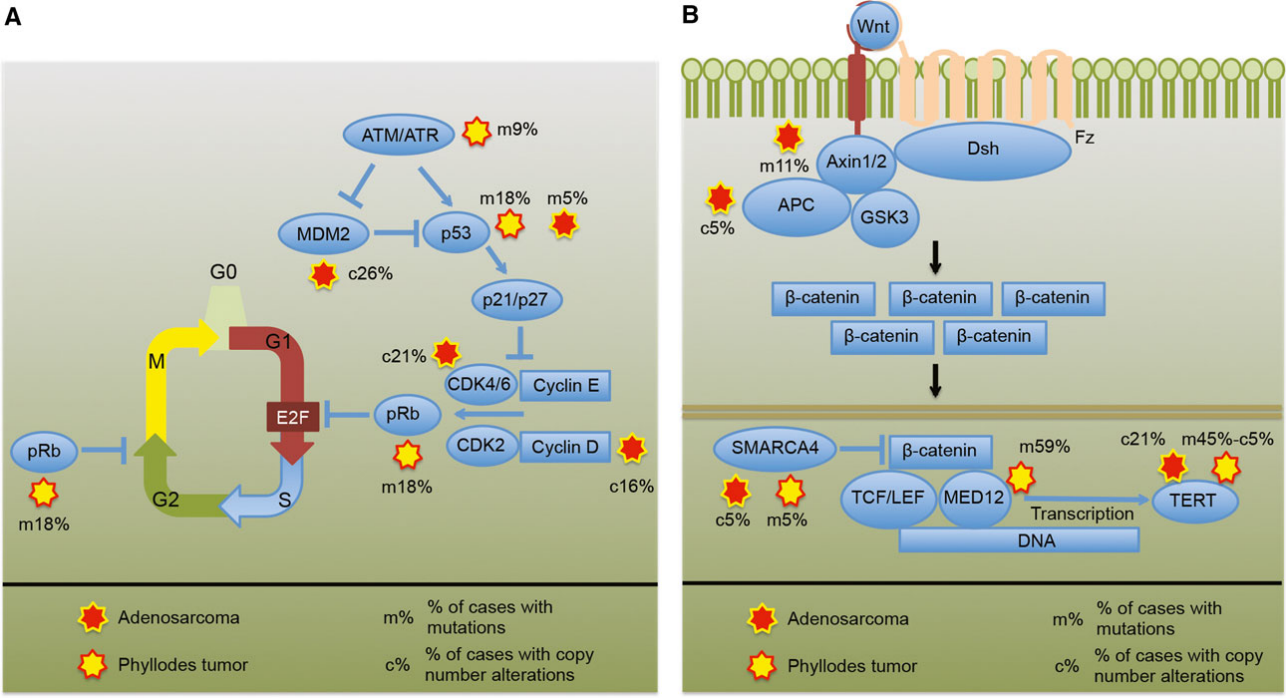
Pathway analysis in uterine adenosarcomas and phyllodes tumors of the breast. (A) p53/pRb/cell cycle‐related pathways. (B) Wnt signaling/nuclear β‐catenin pathways. Pathway members affected by genetic alterations in uterine adenosarcomas (red stars) and phyllodes tumors (yellow stars) are highlighted. The prevalence of cases affected by mutations (m) or copy number alterations (c) within each histologic type is displayed next to the yellow or red stars.

Given that uterine adenosarcomas and PTs of the breast seem to differ in their repertoire of genetic alterations, we performed a pathway analysis to define whether the nonsynonymous somatic mutations, amplifications, and homozygous deletions observed in these two entities would converge into the activation of similar molecular pathways (Table S3, Fig. 5). Significantly altered pathways in adenosarcomas were those associated with p53 and pRb signaling, largely due to amplifications of *MDM2*/*CDK4* (Fig. 5A). In addition, the Wnt signaling pathway was significantly altered in adenosarcomas, which harbored, among other alterations in Wnt pathway‐related genes (Barker *et al*., 2001), homozygous deletions of *APC* (1/19, 5%) and *SMARCA4* (1/19, 5%) and nonsynonymous mutations affecting *AXIN1* (1/19, 5%) and *AXIN2* (1/19, 5%) (Fig. 5B). Paralleling the high prevalence of *TERT* promoter mutations in PTs, a telomere‐associated pathway was significantly associated with this lesion type. Moreover, PTs were significantly enriched for somatic genetic alterations affecting genes in growth factor receptor‐, PI3K‐, and cell cycle‐related pathways (Fig. 5A). Enrichment for these pathways in our cohort of PTs was largely due to the high prevalence of alterations affecting *bona fide* cancer genes (e.g., *TP53*,* RB1*,* EGFR*,* ERBB2*,* ERBB3*,* PIK3CA*) in the malignant and borderline tumors, given that similar results were obtained for those when we repeated the analysis stratifying PTs by grade. Interestingly, benign and borderline PTs were significantly enriched for somatic genetic alterations associated with the β‐catenin nuclear pathway, which is a component of the Wnt pathway. This enrichment was driven by mutations in *MED12* (11/12 benign and borderline cases, 92%), *TERT* promoter (5/12, 42%), and *SMARCA4* (1/12, 8%; Fig. 5B), genes that have been linked to the Wnt pathway (Barker *et al*., 2001; Friedman, 2011; Rocha *et al*., 2010). PTs show indeed immunohistochemical nuclear accumulation of β‐catenin in stromal cells, which is more frequent in benign than in malignant tumors (Lacroix‐Triki *et al*., 2010; Sawyer *et al*., 2002). Therefore, our pathway analysis depicted some overlap, although incomplete, between adenosarcomas and PTs, which may partially explain their morphologic similarities.

## 4 Discussion

**Figure 6:**
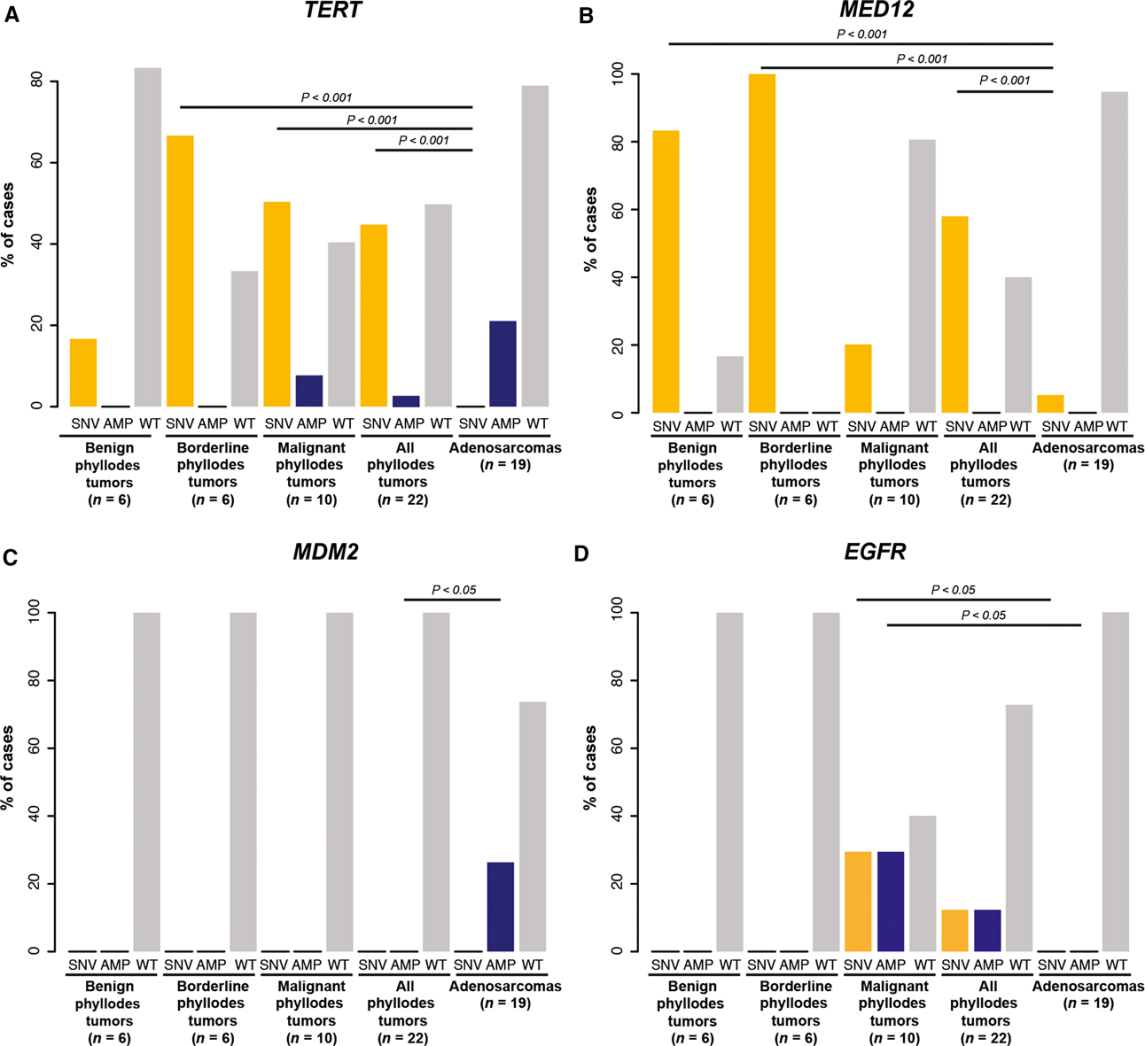
Comparison of the prevalence of genetic alterations affecting selected genes in uterine adenosarcomas and phyllodes tumors of the breast. (A) *TERT*, (B) *MED12*, (C) *MDM2*. D) *EGFR*. AMP, amplification; SNV, single nucleotide variant; WT, wild‐type.

Here, we demonstrate that uterine adenosarcomas and PTs of the breast differ in their highly recurrently altered genes (Fig. 6). The single gene altered in two or more cases of both groups was *TERT*. Importantly, however, the prevalence of genetic alterations affecting *TERT* was higher in PTs than in adenosarcomas, and the mechanism by which *TERT* was altered differed in these lesions. While *TERT* was preferentially altered by hotspot gene promoter mutations in PTs (Cani *et al*., 2015; Liu *et al*., 2016; Piscuoglio *et al*., 2016b; Yoshida *et al*., 2015), only *TERT* gene amplification events were detected in the adenosarcomas studied here (Fig. 6A). Although these findings suggest that telomerase activation plays a role in cell immortalization in both entities, *TERT* is commonly altered in a variety of cancers (Vinagre *et al*., 2013). Hence, in this context, despite the presence of *TERT* alterations in both PTs and adenosarcomas, these findings should not necessarily be interpreted as evidence of a genotypic–phenotypic correlation and of genetic similarities between these tumor types. Exon 2 *MED12* mutations, which are founder clonal mutations in a large subset of PTs (Piscuoglio *et al*., 2016b; Tan *et al*., 2015), were not present in adenosarcomas (Fig. 6B). Conversely, *MDM2*/*CDK4* amplifications, a consistent finding in approximately a quarter of adenosarcomas (Howitt *et al*., 2015; Piscuoglio *et al*., 2016a), were absent in PTs (Fig. 6C). In addition, genetic alterations affecting several *bona fide* cancer genes, including mutations and amplifications in *EGFR* (Fig. 6D), were more common in PTs, particularly in malignant lesions.

Despite these differences at the gene level, both PTs and adenosarcomas appear to have enrichment for somatic genetic alterations affecting genes pertaining to four main pathways: p53/MDM2, pRb/CDK4, PI3K, and Wnt/β‐catenin signaling pathways. Our pathway analysis indeed revealed that the phenotypic similarities between adenosarcomas and PTs could be a result of the activation of similar molecular pathways (convergent phenotype) through distinct somatic genetic alterations (Weigelt and Reis‐Filho, 2014). In addition to distinct somatic genetic alterations likely resulting in TERT activation in PTs and adenosarcomas, both tumor types displayed activation of Wnt signaling, as well as enrichment for alterations in p53/pRb/cell cycle‐related pathways (Fig. 5). The latter was mainly affected by *MDM2*/*CDK4*,* CCND2*, and *CCND3* amplifications in adenosarcomas and by mutations in *TP53*,* RB1*,* ATM*, and *ATR*, among others, in PTs. A significant enrichment for genetic alterations in PI3K‐related pathways was only detected in PTs (Table S3); however, mutations affecting PI3K pathway members have also been observed in adenosarcomas. Howitt *et al*. (2015) previously described that PI3K pathway members were affected in up to 72% of adenosarcomas. In our cohort of adenosarcomas, we detected alterations in PI3K pathway in 26% (5/19) of cases (*PIK3CA*,* PIK3CG*,* PIK3R1*,* PTEN* mutations and/or amplification of *AKT2* or *ERBB3*). In PTs of all grades, mutations in PI3K pathway‐related genes occur at a lower frequency (14% of 22 cases here described), whereas in malignant cases, they have been found to be more frequent (Liu *et al*., 2016; Piscuoglio *et al*., 2016b; Tan *et al*., 2015). Of 13 malignant PTs analyzed by Piscuoglio *et al*. (2016b), five (38%) displayed mutations in PI3K pathway‐related genes (*PIK3CA*,* PDGFRB*,* PTEN*,* AKT1*,* MTOR*,* ERBB2*, and/or *ERBB3* mutations). Additional previous studies described *PIK3CA* mutations in 5% of 79 PTs including all grades (Tan *et al*., 2015), and in three of 10 (30%) malignant PTs (Liu *et al*., 2016).

In agreement with the genetic heterogeneity previously described across benign and malignant PTs (Piscuoglio *et al*., 2016b; Tan *et al*., 2015), the significant genetic differences between adenosarcomas and PTs vary according to the grade of PTs. While benign PTs differ from adenosarcomas by the presence of *MED12* mutations, malignant PTs differ from adenosarcomas by a significantly higher rate of mutations and/or amplifications affecting *bona fide* cancer genes, such as *TP53*,* RB1*,* EGFR*, and *TERT*. Interestingly, in PTs, the number of both mutations and copy number alterations increases according to tumor grade (Fig. 2), whereas an increased number of copy number alterations but not of single nucleotide variants has been reported in adenosarcomas with stromal overgrowth (Howitt *et al*., 2015), suggesting that the genetic mechanisms of progression also differ between adenosarcomas and PTs. A common histologic feature indicative of a more aggressive behavior in adenosarcomas and PTs is the presence of stromal overgrowth and stromal heterologous components. Notably, we found a potential association between rhabdomyoblastic differentiation in adenosarcomas and *DICER1* mutations (Piscuoglio *et al*., 2016a), which occur in familial and sporadic forms of embryonal rhabdomyosarcomas (Doros *et al*., 2012). By contrast, liposarcomatous differentiation in PTs is not underpinned by amplification of the *MDM2/CDK4* locus (Liu *et al*., 2016; Lyle *et al*., 2016), which is a well‐characterized driver of well‐differentiated and dedifferentiated liposarcomas and frequently detected in uterine adenosarcomas (Howitt *et al*., 2015; Piscuoglio *et al*., 2016a). Stromal overgrowth in uterine adenosarcomas has been reported by Howitt *et al*. (2015) to be associated with higher levels of gene copy number alterations compared to those without stromal overgrowth. In our study (Piscuoglio *et al*., 2016a), no associations between stromal overgrowth and specific genetic alterations were detected. Additional studies to define the drivers of adenosarcomas and PTs with heterologous elements and stromal overgrowth are warranted.

Our study has several limitations. Due to the rarity of adenosarcomas and PTs, the relatively small number of cases may have limited our ability to identify the differences between uterine adenosarcomas and PTs. Importantly, given the limited number of cases within some subsets of PTs and adenosarcomas when stratified according to grade, the results obtained in the comparisons between the subsets of PTs and adenosarcomas stratified according to grade should be perceived as exploratory and hypothesis‐generating. It should be noted, however, that despite the small sample size, our study has revealed qualitative and quantitative differences in their repertoire of somatic genetic alterations found in these tumor types. In addition, our sequencing analysis is limited to the genes targeted in our panel. It is possible that whole‐genome, whole‐exome, or RNA‐sequencing experiments would result in additional similarities or more overt differences between adenosarcomas and PTs. In our previous analysis of adenosarcomas, RNA‐sequencing revealed, for instance, fusion genes involving *ESR1* and *NCOA* family members in two of six samples (Piscuoglio *et al*., 2016a). No RNA‐sequencing data on PT were available for comparison; however, to the best of our knowledge, recurrent fusion genes have not been described in PTs or in uterine adenosarcomas. Third, our initial hypothesis was that adenosarcomas and PTs would constitute an example of a genotypic–phenotypic correlation regardless of site of origin, akin to adenoid cystic (Martelotto *et al*., 2015; Persson *et al*., 2009) and mucoepidermoid carcinomas (O'Neill, 2009). Given that no highly recurrent/pathognomonic somatic genetic alteration underpinning both tumor types was identified, a substantially larger study to characterize the genomic differences between uterine adenosarcomas and PTs is warranted. The initiation of international and multi‐institutional consortia for the prospective collection of fresh frozen samples of large cohorts of PTs and adenosarcomas, enabling a more comprehensive genetic characterization of these rare tumor types, is warranted.

Despite these limitations, our analysis revealed that although PTs and adenosarcomas differ quantitatively and qualitatively at the gene level, both lesions are enriched for somatic genetic alterations affecting Wnt and/or the β‐catenin nuclear signaling pathway‐related genes. Furthermore, adenosarcomas and PTs harbor somatic genetic alterations affecting *TERT*, the canonical genes of the p53 pathway (e.g., *TP53* somatic mutations often coupled with loss of heterozygosity (LOH) of the wild‐type allele in 18% of PTs, and *MDM2* amplification in 26% of adenosarcomas), G1/S checkpoint‐related genes (e.g., *RB1* mutations often coupled with LOH of the wild‐type allele in 18% of PTs, and *CDK4*,* CCND2*, and/or *CCND3* amplifications in 26% of adenosarcomas), and PI3K pathway‐related genes (e.g., *PIK3CA* mutations in 5% of PTs and *PIK3CA*,* PTE*N, and/or *PIK3R1* mutations and/or *AKT2* amplifications in 21% of adenosarcomas). The lack of an overt genotypic–phenotypic correlation between adenosarcomas and PTs may stem from the fact that similar pathways are affected in both lesions but through distinct genetic alterations. The alternative hypothesis, however, is that the phenotypic similarity would not have a direct genetic basis. Given that in adenosarcomas and PTs, the mesenchymal and epithelial components are not clonally related and that the latter may not be neoplastic (Piscuoglio *et al*., 2016a,b; Tan *et al*., 2015), the histologic similarities may actually result from a similar pattern of interaction between neoplastic stromal cells and hyperplastic epithelial cells. In metastatic lesions of adenosarcomas and PTs, the biphasic architecture is rarely observed (McCluggage, 2010; Tan *et al*., 2016); thus, the epithelial–stromal interaction is likely dependent on local/paracrine factors and/or, once the lesion has acquired metastatic potential, the mesenchymal cells become independent from stimulus from the epithelium to proliferate. Some studies have suggested that in PTs Wnt factors secreted by the epithelium may activate Wnt signaling and proliferation in the stromal cells, which accumulate β‐catenin in the nucleus (Karim *et al*., 2009; Sawhney *et al*., 1992; Sawyer *et al*., 2002). Notably, benign and borderline PTs, as well as adenosarcomas, were significantly associated with β‐catenin nuclear/Wnt signaling pathway. *MED12* mutations are likely to cooperate for this activation in PTs, given that *MED12* has been shown to be essential for canonical Wnt signaling (Rocha *et al*., 2010). Finally, given that both the endometrial mucosa and the terminal duct‐lobular units of the breast are composed of estrogen‐responsive epithelial and stromal cells, hormonal factors may play a role in the epithelial–stromal interaction of both entities. Additional studies comparing the epithelial–stromal interaction in uterine adenosarcomas and PTs of the breast are warranted.

## Author contributions

JSR‐F and BW conceived and designed the study. KAB, CKYN, and PS performed bioinformatics analyses. FCG, KAB, SP, CKYN, ADP, CM, PS, ME, MPM, EB, RAS, BPR, LN, JSR‐F, and BW interpreted the data. JSR‐F and BW supervised the study. FCG wrote the first draft, which was revised by JSR‐F and BW. All authors edited and approved the final draft of the manuscript.

## Supporting information


**Fig. S1.** Amplifications and homozygous deletions identified by massively parallel sequencing in uterine adenosarcomas and phyllodes tumors of the breast included in this study.

**Fig. S2.** Comparisons of nonsynonymous somatic mutations detected by massively parallel sequencing in uterine adenosarcomas and phyllodes tumors of the breast stratified by grade.

**Fig. S3.** Comparisons of the frequency of copy number alterations identified in uterine adenosarcomas and phyllodes tumors of the breast stratified by grade.

**Fig. S4.** Comparisons of nonsynonymous somatic mutations detected by massively parallel sequencing in uterine adenosarcomas stratified by grade and phyllodes tumors of the breast.

**Fig. S5.** Comparisons of the frequency of copy number alterations identified in uterine adenosarcomas stratified by grade and phyllodes tumors of the breast.

**Table S1.** 341 genes concurrently present on all massively parallel sequencing platforms previously used to analyze the uterine adenosarcomas (*n* = 19) and phyllodes tumors of the breast (*n* = 22) included in this study.

**Table S2.** List of somatic mutations identified by massively parallel sequencing in uterine adenosarcomas (*n* = 19) and phyllodes tumors of the breast (*n* = 22) included in this study.

**Table S3.** Pathway analysis using gProfiler, MsigDB and DAVID in uterine adenosarcomas and phyllodes tumors of the breast included in this study.
