# Supplementary figures and images for: Genetic analysis of uterine adenosarcomas and phyllodes tumors of the breast
Source: Mol Oncol. 2017 May 16;11(8):913–26. doi: 10.1002/1878-0261.12049 (PMC5537914; doi:10.1002/1878-0261.12049)

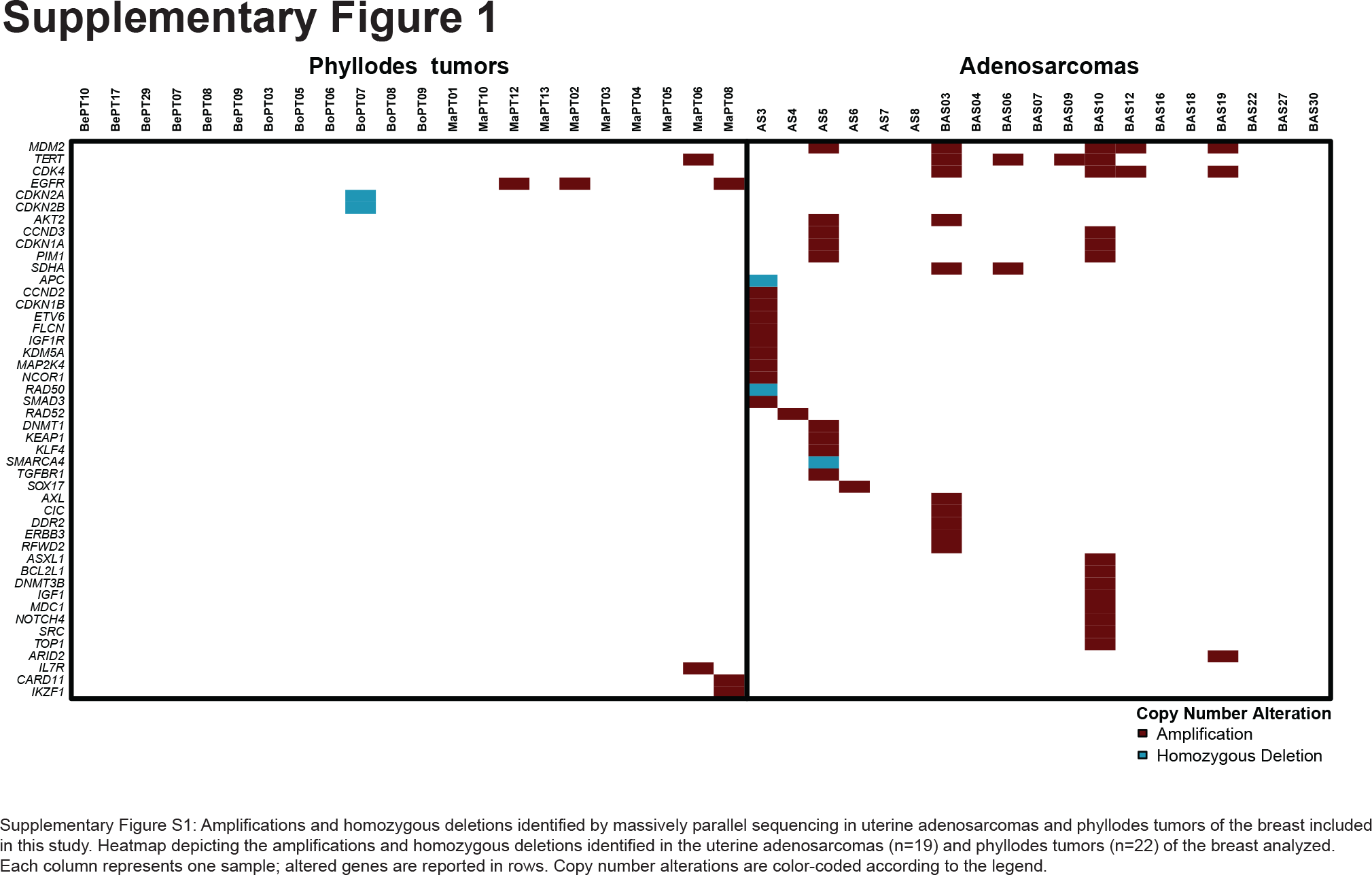

Supplement: Supplementary file 1 — Fig. S1. Amplifications and homozygous deletions identified by massively parallel sequencing in uterine adenosarcomas and phyllodes tumors of the breast included in this study. [file MOL2-11-913-s001.tif]

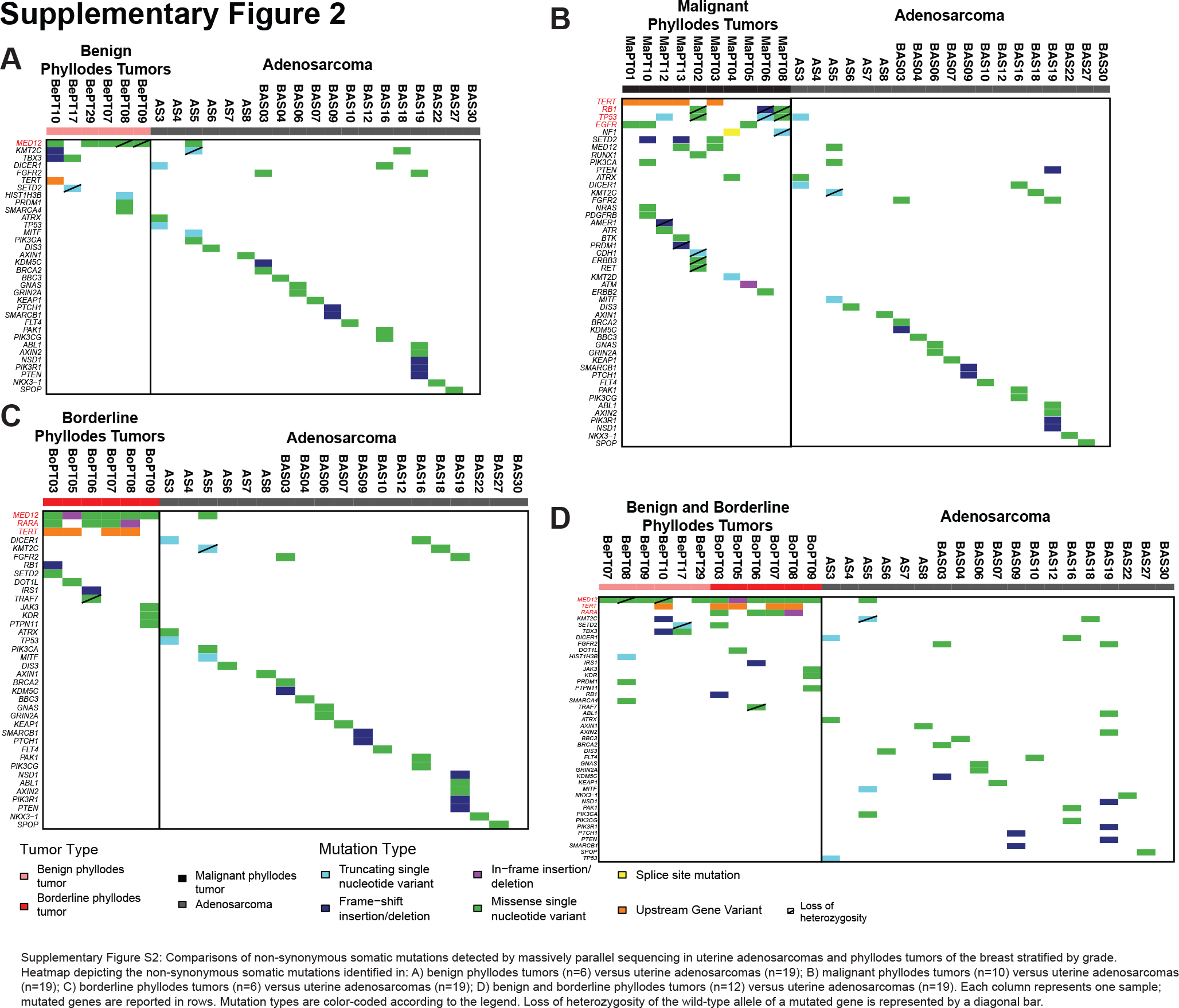

Supplement: Supplementary file 2 — Fig. S2. Comparisons of nonsynonymous somatic mutations detected by massively parallel sequencing in uterine adenosarcomas and phyllodes tumors of the breast stratified by grade. [file MOL2-11-913-s002.tif]

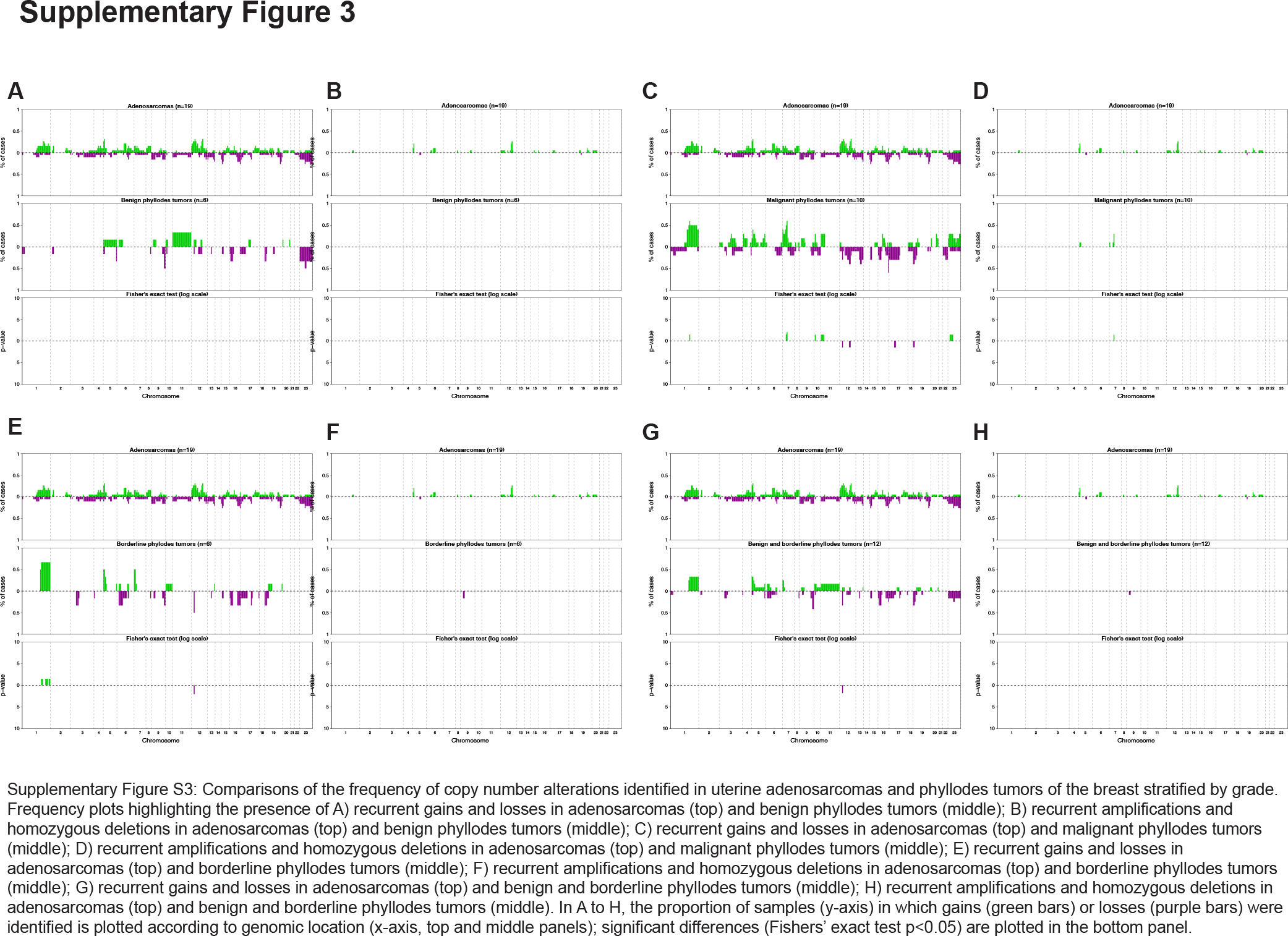

Supplement: Supplementary file 3 — Fig. S3. Comparisons of the frequency of copy number alterations identified in uterine adenosarcomas and phyllodes tumors of the breast stratified by grade. [file MOL2-11-913-s003.tif]

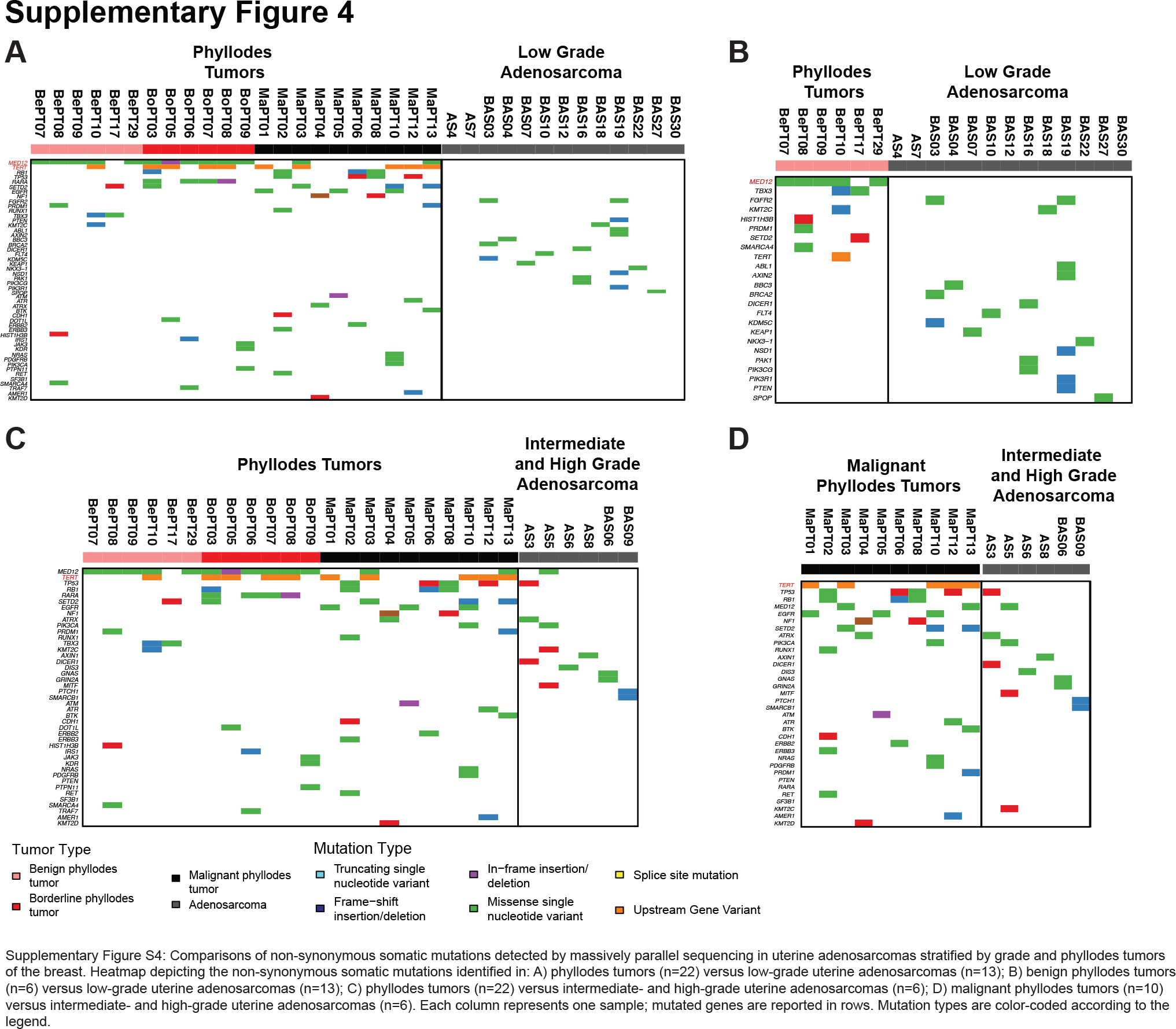

Supplement: Supplementary file 4 — Fig. S4. Comparisons of nonsynonymous somatic mutations detected by massively parallel sequencing in uterine adenosarcomas stratified by grade and phyllodes tumors of the breast. [file MOL2-11-913-s004.tif]

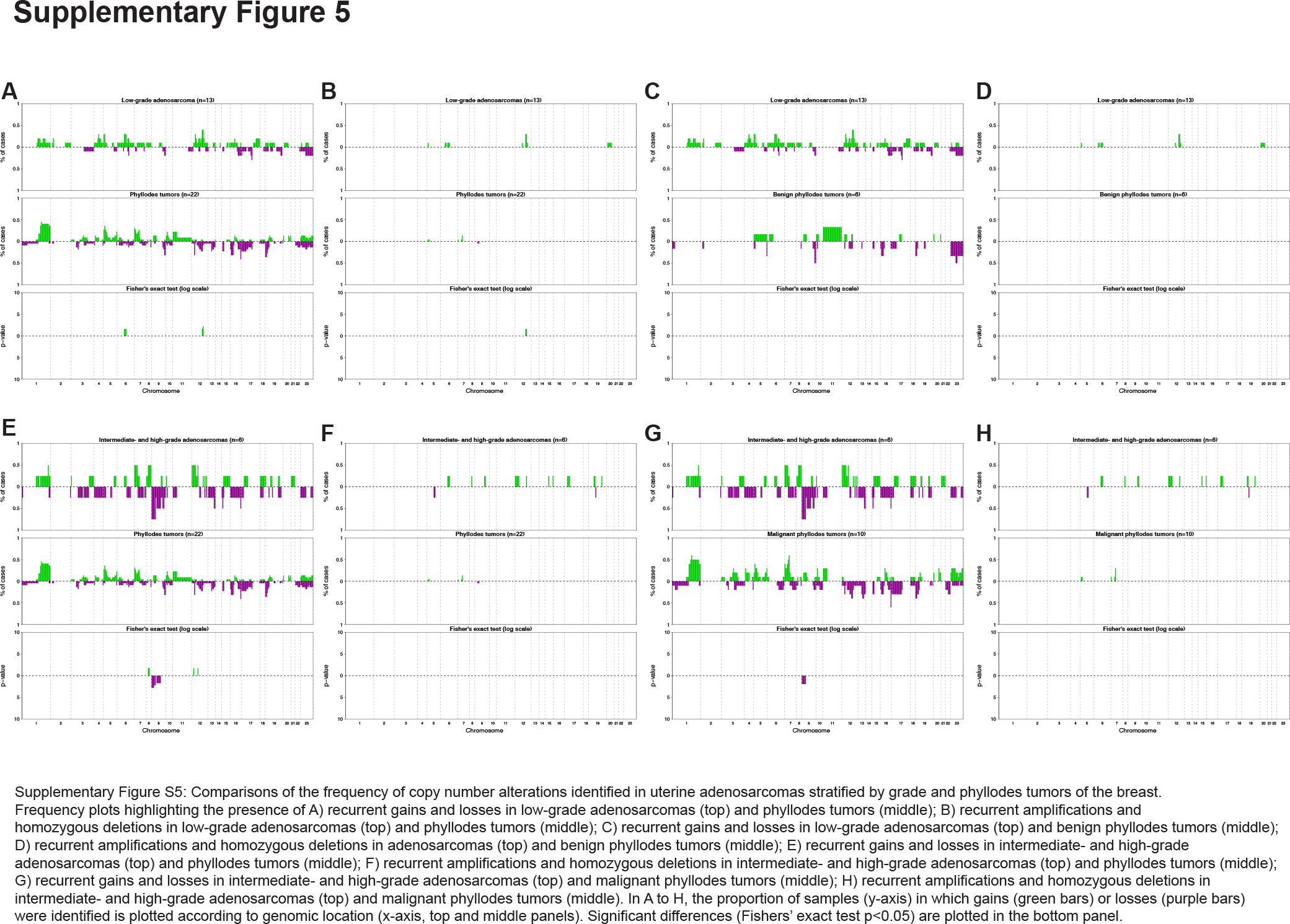

Supplement: Supplementary file 5 — Fig. S5. Comparisons of the frequency of copy number alterations identified in uterine adenosarcomas stratified by grade and phyllodes tumors of the breast. [file MOL2-11-913-s005.tif]
