# Supplementary material for: Genetic analysis of uterine adenosarcomas and phyllodes tumors of the breast
Source: Mol Oncol. 2017 May 16;11(8):913–26. doi: 10.1002/1878-0261.12049 (PMC5537914; doi:10.1002/1878-0261.12049)
Supplement: Supplementary file 6 — Table S1. 341 genes concurrently present on all massively parallel sequencing platforms previously used to analyze the uterine adenosarcomas (n = 19) and phyllodes tumors of the breast (n = 22) included in this study. [file MOL2-11-913-s006.pdf]

Supplementary Table S1: List of 341 genes present in the massively parallel sequencing platforms previously used to analyzed the uterine adenosarcomas and phyllodes tumors of the breast included in this study	
**HGNC symbol**	**Approved Name**
ABL1	c-abl oncogene 1, non-receptor tyrosine kinase
AKT1	v-akt murine thymoma viral oncogene homolog 1
AKT2	v-akt murine thymoma viral oncogene homolog 2
AKT3	v-akt murine thymoma viral oncogene homolog 3
ALK	anaplastic lymphoma receptor tyrosine kinase
ALOX12B	arachidonate 12-lipoxygenase, 12R type
AMER1	family with sequence similarity 123B
APC	adenomatous polyposis coli
AR	androgen receptor
ARAF	v-raf murine sarcoma 3611 viral oncogene homolog
ARID1A	AT rich interactive domain 1A (SWI-like)
ARID1B	AT rich interactive domain 1B (SWI1-like)
ARID2	AT rich interactive domain 2 (ARID, RFX-like)
ARID5B	AT rich interactive domain 5B (MRF1-like)
ASXL1	additional sex combs like 1 (Drosophila)
ASXL2	additional sex combs like 2 (Drosophila)
ATM	ataxia telangiectasia mutated
ATR	ataxia telangiectasia and Rad3 related
ATRX	alpha thalassemia/mental retardation syndrome X-linked
AURKA	aurora kinase A
AURKB	aurora kinase B
AXIN1	axin 1
AXIN2	axin 2
AXL	AXL receptor tyrosine kinase
B2M	beta-2-microglobulin
BAP1	BRCA1 associated protein-1 (ubiquitin carboxy-terminal hydrolase)
BARD1	BRCA1 associated RING domain 1
BBC3	BCL2 binding component 3
BCL2	B-cell CLL/lymphoma 2
BCL2L1	BCL2-like 1
BCL2L11	BCL2-like 11 (apoptosis facilitator)
BCL6	B-cell CLL/lymphoma 6
BCOR	BCL6 corepressor
BLM	Bloom syndrome, RecQ helicase-like
BMPR1A	bone morphogenetic protein receptor, type IA
BRAF	v-raf murine sarcoma viral oncogene homolog B
BRCA1	breast cancer 1, early onset
BRCA2	breast cancer 2, early onset
BRD4	bromodomain containing 4
BRIP1	BRCA1 interacting protein C-terminal helicase 1
BTK	Bruton agammaglobulinemia tyrosine kinase
CARD11	caspase recruitment domain family, member 11
CASP8	caspase 8, apoptosis-related cysteine peptidase
CBFB	core-binding factor, beta subunit
CBL	Cas-Br-M (murine) ecotropic retroviral transforming sequence
CCND1	cyclin D1
CCND2	cyclin D2
CCND3	cyclin D3
CCNE1	cyclin E1
CD274	CD274 molecule
CD276	CD276 molecule
CD79B	CD79b molecule, immunoglobulin-associated beta
CDC73	cell division cycle 73, Paf1/RNA polymerase II complex component, homolog (S. cerevisiae)
CDH1	cadherin 1, type 1, E-cadherin (epithelial)
CDK12	cyclin-dependent kinase 12
CDK4	cyclin-dependent kinase 4
CDK6	cyclin-dependent kinase 6
CDK8	cyclin-dependent kinase 8
CDKN1A	cyclin-dependent kinase inhibitor 1A (p21, Cip1)
CDKN1B	cyclin-dependent kinase inhibitor 1B (p27, Kip1)
CDKN2A	cyclin-dependent kinase inhibitor 2A
CDKN2B	cyclin-dependent kinase inhibitor 2B (p15, inhibits CDK4)
CDKN2C	cyclin-dependent kinase inhibitor 2C (p18, inhibits CDK4)
CHEK1	checkpoint kinase 1
CHEK2	checkpoint kinase 2
CIC	capicua homolog (Drosophila)
CREBBP	CREB binding protein
CRKL	v-crk sarcoma virus CT10 oncogene homolog (avian)-like
CRLF2	cytokine receptor-like factor 2
CSF1R	colony stimulating factor 1 receptor
CTCF	CCCTC-binding factor (zinc finger protein)
CTLA4	cytotoxic T-lymphocyte-associated protein 4
CTNNB1	catenin (cadherin-associated protein), beta 1, 88kDa
CUL3	cullin 3
DAXX	death-domain associated protein
DCUN1D1	DCN1, defective in cullin neddylation 1, domain containing 1 (S. cerevisiae)
DDR2	discoidin domain receptor tyrosine kinase 2
DICER1	dicer 1, ribonuclease type III
DIS3	DIS3 mitotic control homolog (S. cerevisiae)
DNMT1	DNA (cytosine-5-)-methyltransferase 1
DNMT3A	DNA (cytosine-5-)-methyltransferase 3 alpha
DNMT3B	DNA (cytosine-5-)-methyltransferase 3 beta
DOT1L	DOT1-like, histone H3 methyltransferase (S. cerevisiae)
E2F3	E2F transcription factor 3
EED	embryonic ectoderm development

HGNC symbol	Approved Name
EGFL7	EGF-like-domain, multiple 7
EGFR	epidermal growth factor receptor
EIF1AX	eukaryotic translation initiation factor 1A, X-linked
EP300	E1A binding protein p300
EPCAM	epithelial cell adhesion molecule
EPHA3	EPH receptor A3
EPHA5	EPH receptor A5
EPHB1	EPH receptor B1
ERBB2	v-erb-b2 avian erythroblastic leukemia viral oncogene homolog 2
ERBB3	v-erb-b2 avian erythroblastic leukemia viral oncogene homolog 3
ERBB4	v-erb-b2 avian erythroblastic leukemia viral oncogene homolog 4
ERCC2	excision repair cross-complementing rodent repair deficiency, complementation group 2
ERCC3	excision repair cross-complementing rodent repair deficiency, complementation group 3
ERCC4	excision repair cross-complementing rodent repair deficiency, complementation group 4
ERCC5	excision repair cross-complementing rodent repair deficiency, complementation group 5
ERG	v-ets erythroblastosis virus E26 oncogene homolog (avian)
ESR1	estrogen receptor 1
ETV1	ets variant 1
ETV6	ets variant 6
EZH2	enhancer of zeste homolog 2 (Drosophila)
FAM175A	family with sequence similarity 175, member A
FAM46C	family with sequence similarity 46, member C
FANCA	Fanconi anemia, complementation group A
FANCC	Fanconi anemia, complementation group C
FAT1	FAT tumor suppressor homolog 1 (Drosophila)
FBXW7	F-box and WD repeat domain containing 7
FGF19	fibroblast growth factor 19
FGF3	fibroblast growth factor 3
FGF4	fibroblast growth factor 4
FGFR1	fibroblast growth factor receptor 1
FGFR2	fibroblast growth factor receptor 2
FGFR3	fibroblast growth factor receptor 3
FGFR4	fibroblast growth factor receptor 4
FH	fumarate hydratase
FLCN	folliculin
FLT1	fms-related tyrosine kinase 1 (vascular endothelial growth factor/vascular permeability factor receptor)
FLT3	fms-related tyrosine kinase 3
FLT4	fms-related tyrosine kinase 4
FOXA1	forkhead box A1
FOXL2	forkhead box L2
FOXP1	forkhead box P1
FUBP1	far upstream element (FUSE) binding protein 1
GATA1	GATA binding protein 1 (globin transcription factor 1)
GATA2	GATA binding protein 2
GATA3	GATA binding protein 3
GNA11	guanine nucleotide binding protein (G protein), alpha 11 (Gq class)
GNAQ	guanine nucleotide binding protein (G protein), q polypeptide
GNAS	GNAS complex locus
GREM1	gremlin 1
GRIN2A	glutamate receptor, ionotropic, N-methyl D-aspartate 2A
GSK3B	glycogen synthase kinase 3 beta
H3F3C	H3 histone, family 3C
HGF	hepatocyte growth factor (hepapoietin A; scatter factor)
HIST1H1C	histone cluster 1, H1c
HIST1H2BD	histone cluster 1, H2bd
HIST1H3B	histone cluster 1, H3b
HNF1A	HNF1 homeobox A
HRAS	Harvey rat sarcoma viral oncogene homolog
ICOSLG	inducible T-cell co-stimulator ligand
IDH1	isocitrate dehydrogenase 1 (NADP+), soluble
IDH2	isocitrate dehydrogenase 2 (NADP+), mitochondrial
IFNGR1	interferon gamma receptor 1
IGF1	insulin-like growth factor 1 (somatomedin C)
IGF1R	insulin-like growth factor 1 receptor
IGF2	insulin-like growth factor 2 (somatomedin A)
IKBKE	inhibitor of kappa light polypeptide gene enhancer in B-cells, kinase epsilon
IKZF1	IKAROS family zinc finger 1 (Ikaros)
IL10	interleukin 10
IL7R	interleukin 7 receptor
INPP4A	inositol polyphosphate-4-phosphatase, type I, 107kDa
INPP4B	inositol polyphosphate-4-phosphatase, type II, 105kDa
INSR	insulin receptor
IRF4	interferon regulatory factor 4
IRS1	insulin receptor substrate 1
IRS2	insulin receptor substrate 2
JAK1	Janus kinase 1
JAK2	Janus kinase 2
JAK3	Janus kinase 3
JUN	jun proto-oncogene
KDM5A	lysine (K)-specific demethylase 5A
KDM5C	lysine (K)-specific demethylase 5C
KDM6A	lysine (K)-specific demethylase 6A
KDR	kinase insert domain receptor (a type III receptor tyrosine kinase)
KEAP1	kelch-like ECH-associated protein 1
KIT	v-kit Hardy-Zuckerman 4 feline sarcoma viral oncogene homolog
KLF4	Kruppel-like factor 4 (gut)
KMT2A	myeloid/lymphoid or mixed-lineage leukemia (trithorax homolog, Drosophila)
KMT2C	lysine (K)-specific methyltransferase 2C

HGNC symbol	Approved Name
KMT2D	lysine (K)-specific methyltransferase 2D
KRAS	Kirsten rat sarcoma viral oncogene homolog
LATS1	LATS, large tumor suppressor, homolog 1 (Drosophila)
LATS2	LATS, large tumor suppressor, homolog 2 (Drosophila)
LMO1	LIM domain only 1 (rhombotin 1)
MAP2K1	mitogen-activated protein kinase kinase 1
MAP2K2	mitogen-activated protein kinase kinase 2
MAP2K4	mitogen-activated protein kinase kinase 4
MAP3K1	mitogen-activated protein kinase kinase kinase 1, E3 ubiquitin protein ligase
MAP3K13	mitogen-activated protein kinase kinase kinase 13
MAPK1	mitogen-activated protein kinase 1
MAX	MYC associated factor X
MCL1	myeloid cell leukemia sequence 1 (BCL2-related)
MDC1	mediator of DNA-damage checkpoint 1
MDM2	MDM2 oncogene, E3 ubiquitin protein ligase
MDM4	Mdm4 p53 binding protein homolog (mouse)
MED12	mediator complex subunit 12
MEF2B	myocyte enhancer factor 2B
MEN1	multiple endocrine neoplasia I
MET	met proto-oncogene
MITF	microphthalmia-associated transcription factor
MLH1	mutL homolog 1, colon cancer, nonpolyposis type 2 (E. coli)
MPL	myeloproliferative leukemia virus oncogene
MRE11A	MRE11 meiotic recombination 11 homolog A (S. cerevisiae)
MSH2	mutS homolog 2, colon cancer, nonpolyposis type 1 (E. coli)
MSH6	mutS homolog 6 (E. coli)
MTOR	mechanistic target of rapamycin (serine/threonine kinase)
MUTYH	mutY homolog (E. coli)
MYC	v-myc myelocytomatosis viral oncogene homolog (avian)
MYCL	v-myc myelocytomatosis viral oncogene homolog 1, lung carcinoma derived (avian)
MYCN	v-myc myelocytomatosis viral related oncogene, neuroblastoma derived (avian)
MYD88	myeloid differentiation primary response gene (88)
MYOD1	myogenic differentiation 1
NBN	nibrin
NCOR1	nuclear receptor corepressor 1
NF1	neurofibromin 1
NF2	neurofibromin 2 (merlin)
NFE2L2	nuclear factor (erythroid-derived 2)-like 2
NKX2-1	NK2 homeobox 1
NKX3-1	NK3 homeobox 1
NOTCH1	notch 1
NOTCH2	notch 2
NOTCH3	notch 3
NOTCH4	notch 4
NPM1	nucleophosmin (nucleolar phosphoprotein B23, numatrin)
NRAS	neuroblastoma RAS viral (v-ras) oncogene homolog
NSD1	nuclear receptor binding SET domain protein 1
NTRK1	neurotrophic tyrosine kinase, receptor, type 1
NTRK2	neurotrophic tyrosine kinase, receptor, type 2
NTRK3	neurotrophic tyrosine kinase, receptor, type 3
PAK1	p21 protein (Cdc42/Rac)-activated kinase 1
PAK7	p21 protein (Cdc42/Rac)-activated kinase 7
PALB2	partner and localizer of BRCA2
PARK2	parkinson protein 2, E3 ubiquitin protein ligase (parkin)
PARP1	poly (ADP-ribose) polymerase 1
PAX5	paired box 5
PBRM1	polybromo 1
PDCD1	programmed cell death 1
PDGFRA	platelet-derived growth factor receptor, alpha polypeptide
PDGFRB	platelet-derived growth factor receptor, beta polypeptide
PDPK1	3-phosphoinositide dependent protein kinase-1
PHOX2B	paired-like homeobox 2b
PIK3C2G	phosphoinositide-3-kinase, class 2, gamma polypeptide
PIK3C3	phosphoinositide-3-kinase, class 3
PIK3CA	phosphatidylinositol-4,5-bisphosphate 3-kinase, catalytic subunit alpha
PIK3CB	phosphatidylinositol-4,5-bisphosphate 3-kinase, catalytic subunit beta
PIK3CD	phosphoinositide-3-kinase, catalytic, delta polypeptide
PIK3CG	phosphoinositide-3-kinase, catalytic, gamma polypeptide
PIK3R1	phosphoinositide-3-kinase, regulatory subunit 1 (alpha)
PIK3R2	phosphoinositide-3-kinase, regulatory subunit 2 (beta)
PIK3R3	phosphoinositide-3-kinase, regulatory subunit 3 (gamma)
PIM1	pim-1 oncogene
PLK2	polo-like kinase 2
PMAIP1	phorbol-12-myristate-13-acetate-induced protein 1
PMS1	PMS1 postmeiotic segregation increased 1 (S. cerevisiae)
PMS2	PMS2 postmeiotic segregation increased 2 (S. cerevisiae)
PNRC1	proline-rich nuclear receptor coactivator 1
POLE	polymerase (DNA directed), epsilon, catalytic subunit
PPP2R1A	protein phosphatase 2, regulatory subunit A, alpha
PRDM1	PR domain containing 1, with ZNF domain
PRKAR1A	protein kinase, cAMP-dependent, regulatory, type I, alpha (tissue specific extinguisher 1)
PTCH1	patched 1
PTEN	phosphatase and tensin homolog
PTPN11	protein tyrosine phosphatase, non-receptor type 11
PTPRD	protein tyrosine phosphatase, receptor type, D
PTPRS	protein tyrosine phosphatase, receptor type, S
PTPRT	protein tyrosine phosphatase, receptor type, T
RAC1	ras-related C3 botulinum toxin substrate 1 (rho family, small GTP binding protein Rac1)

HGNC symbol	Approved Name
RAD50	RAD50 homolog (S. cerevisiae)
RAD51	RAD51 recombinase
RAD51B	RAD51 paralog B
RAD51C	RAD51 paralog C
RAD51D	RAD51 paralog D
RAD52	RAD52 homolog (S. cerevisiae)
RAD54L	RAD54-like (S. cerevisiae)
RAF1	v-raf-1 murine leukemia viral oncogene homolog 1
RARA	retinoic acid receptor, alpha
RASA1	RAS p21 protein activator (GTPase activating protein) 1
RB1	retinoblastoma 1
RBM10	RNA binding motif protein 10
RECQL4	RecQ protein-like 4
REL	v-rel reticuloendotheliosis viral oncogene homolog (avian)
RET	ret proto-oncogene
RFWD2	ring finger and WD repeat domain 2
RHOA	ras homolog gene family, member A
RICTOR	RPTOR independent companion of MTOR, complex 2
RIT1	Ras-like without CAAX 1
RNF43	ring finger protein 43
ROS1	c-ros oncogene 1 , receptor tyrosine kinase
RPS6KA4	ribosomal protein S6 kinase, 90kDa, polypeptide 4
RPS6KB2	ribosomal protein S6 kinase, 70kDa, polypeptide 2
RPTOR	regulatory associated protein of MTOR, complex 1
RUNX1	runt-related transcription factor 1
RYBP	RING1 and YY1 binding protein
SDHA	succinate dehydrogenase complex, subunit A, flavoprotein (Fp)
SDHAF2	succinate dehydrogenase complex assembly factor 2
SDHB	succinate dehydrogenase complex, subunit B, iron sulfur (Ip)
SDHC	succinate dehydrogenase complex, subunit C, integral membrane protein, 15kDa
SDHD	succinate dehydrogenase complex, subunit D, integral membrane protein
SETD2	SET domain containing 2
SF3B1	splicing factor 3b, subunit 1, 155kDa
SH2D1A	SH2 domain containing 1A
SHQ1	SHQ1 homolog (S. cerevisiae)
SMAD2	SMAD family member 2
SMAD3	SMAD family member 3
SMAD4	SMAD family member 4
SMARCA4	SWI/SNF related, matrix associated, actin dependent regulator of chromatin, subfamily a, member 4
SMARCB1	SWI/SNF related, matrix associated, actin dependent regulator of chromatin, subfamily b, member 1
SMARCD1	SWI/SNF related, matrix associated, actin dependent regulator of chromatin, subfamily d, member 1
SMO	smoothened, frizzled family receptor
SOCS1	suppressor of cytokine signaling 1
SOX17	SRY (sex determining region Y)-box 17
SOX2	SRY (sex determining region Y)-box 2
SOX9	SRY (sex determining region Y)-box 9
SPEN	spen homolog, transcriptional regulator (Drosophila)
SPOP	speckle-type POZ protein
SRC	v-src sarcoma (Schmidt-Ruppin A-2) viral oncogene homolog (avian)
STAG2	stromal antigen 2
STK11	serine/threonine kinase 11
STK40	serine/threonine kinase 40
SUFU	suppressor of fused homolog (Drosophila)
SUZ12	suppressor of zeste 12 homolog (Drosophila)
SYK	spleen tyrosine kinase
TBX3	T-box 3
TERT	telomerase reverse transcriptase
TET1	tet oncogene 1
TET2	tet oncogene family member 2
TGFBR1	transforming growth factor, beta receptor 1
TGFBR2	transforming growth factor, beta receptor II (70/80kDa)
TMEM127	transmembrane protein 127
TMPRSS2	transmembrane protease, serine 2
TNFAIP3	tumor necrosis factor, alpha-induced protein 3
TNFRSF14	tumor necrosis factor receptor superfamily, member 14 (herpesvirus entry mediator)
TOP1	topoisomerase (DNA) I
TP53	tumor protein p53
TP63	tumor protein p63
TRAF7	TNF receptor-associated factor 7
TSC1	tuberous sclerosis 1
TSC2	tuberous sclerosis 2
TSHR	thyroid stimulating hormone receptor
U2AF1	U2 small nuclear RNA auxiliary factor 1
VHL	von Hippel-Lindau tumor suppressor
VTCN1	V-set domain containing T cell activation inhibitor 1
WT1	Wilms tumor 1
XIAP	X-linked inhibitor of apoptosis
XPO1	exportin 1 (CRM1 homolog, yeast)
YAP1	Yes-associated protein 1
YES1	v-yes-1 Yamaguchi sarcoma viral oncogene homolog 1
