# Supplementary material for: Genetic analysis of uterine adenosarcomas and phyllodes tumors of the breast
Source: Mol Oncol. 2017 May 16;11(8):913–26. doi: 10.1002/1878-0261.12049 (PMC5537914; doi:10.1002/1878-0261.12049)
Supplement: Supplementary file 7 — Table S2. List of somatic mutations identified by massively parallel sequencing in uterine adenosarcomas (n = 19) and phyllodes tumors of the breast (n = 22) included in this study. [file MOL2-11-913-s007.pdf]

Supplementary Table S2: List of mutations identified by massivelly parallel sequencing in uterine adenosarcomas (n=19) and phyllodes tumors of the breast (n=22)

Sample ID	Gene	Amino Acid Change	Effect	Chromosome	Pos	Ref	Alt	Tumor Depth	Tumor MAF	LOH	CCF	Probabilty Mutation is clonal	95% Confidence Interval (Low)	Clonality	Mutation Taster	CHASM	FATHMM	PROVEAN	Pathogenicity	Kandoth	Lawrence	Cancer Gene Census
BePT07	MED12	p.Gly44Ser	missense_variant	X	70339253	G	A	429	0.17	.	1	0.8692	0.8702	Clonal	D	Passenger	PASSENGER/OTHER	.	Non-pathogenic	.	TRUE	TRUE
BePT08	HIST1H3B	p.Leu61*	stop_gained	6	26032107	A	T	774	0.15	.	0.96	0.8157	0.7931	Clonal	D	.	.	.	Likely_pathogenic	.	TRUE	TRUE
BePT08	MED12	p.Gly44Ser	missense_variant	X	70339253	G	A	598	0.22	LOH	1	0.9419	0.8980	Clonal	D	Passenger	PASSENGER/OTHER	.	Non-pathogenic	.	TRUE	TRUE
BePT08	PRDM1	p.Lys638Asn	missense_variant	6	106554797	G	C	277	0.1	.	0.63	0.0397	0.4360	.	D	Passenger	PASSENGER/OTHER	.	Non-pathogenic	.	TRUE	TRUE
BePT08	SMARCA4	p.Pro1297Leu	missense_variant	19	11144815	C	T	419	0.15	.	0.92	0.7318	0.7129	Clonal	D	Passenger	CANCER	.	Likely_pathogenic	.	TRUE	TRUE
BePT09	MED12	p.Gly44Ser	missense_variant	X	70339253	G	A	578	0.29	.	1	0.8620	0.9218	Clonal	D	Passenger	PASSENGER/OTHER	.	Non-pathogenic	.	TRUE	TRUE
BePT10	KMT2C	p.Leu1330fs	frameshift_variant	7	151900121	TA	T	378	0.18	.	1	0.8765	0.7843	Clonal	A	.	.	.	Likely_pathogenic	TRUE	TRUE	TRUE
BePT10	MED12	p.Gly44Val	missense_variant	X	70339254	G	T	635	0.21	LOH	0.98	0.9134	0.8231	Clonal	D	Passenger	PASSENGER/OTHER	.	Non-pathogenic	.	TRUE	TRUE
BePT10	TBX3	p.Asn296fs	frameshift_variant	12	115115438	GT	G	916	0.24	.	1	0.0028	0.9517	Clonal	A	.	.	.	Likely_pathogenic	TRUE	TRUE	.
BePT10	TERT	c.-59C>T	upstream_gene_variant	5	1295228	G	A	180	0.19	.	1	0.8595	0.7304	Clonal	.	.	.	.	Likely_pathogenic	.	.	TRUE
BePT17	SETD2	p.Gln1273*	stop_gained	3	47162309	G	A	360	0.4	LOH	1	0.9776	0.9600	Clonal	A	.	.	.	Likely_pathogenic	TRUE	TRUE	TRUE
BePT17	SPEN	p.Lys816Lys	synonymous_variant	1	16255183	G	A	561	0.22	.	1	0.9167	0.8589	Clonal	.	.	.	.	Non-pathogenic	.	TRUE	.
BePT17	TBX3	p.Ile294Thr	missense_variant	12	115115445	A	G	475	0.11	.	0.51	0.0000	0.3886	.	D	Passenger	PASSENGER/OTHER	.	Non-pathogenic	TRUE	TRUE	.
BePT29	MED12	p.Gly44Val	missense_variant	X	70339254	G	T	794	0.22	.	0.99	0.8880	0.8493	Clonal	D	.	PASSENGER/OTHER	.	Non-pathogenic	.	TRUE	TRUE
BoPT03	MED12	p.Glu172Lys	missense_variant	X	70339981	G	A	489	0.28	.	1	0.5058	0.9214	Clonal	D	Passenger	PASSENGER/OTHER	.	Non-pathogenic	.	TRUE	TRUE
BoPT03	RARA	p.Cys235Trp	missense_variant	17	38508657	C	G	494	0.31	.	1	0.0047	0.9410	Clonal	D	Passenger	PASSENGER/OTHER	.	Non-pathogenic	.	.	TRUE
BoPT03	RB1	p.Gly310fs	frameshift_variant	13	48939095	TG	T	286	0.13	.	0.58	0.0010	0.4194	.	A	.	.	.	Likely_pathogenic	TRUE	TRUE	TRUE
BoPT03	RB1	p.Ile848Arg	missense_variant	13	49050859	T	G	182	0.08	.	0.35	0.0000	0.2090	.	D	Passenger	CANCER	.	Likely_pathogenic	TRUE	TRUE	TRUE
BoPT03	RB1	p.Pro250fs	frameshift_variant	13	48936979	ACC	A	495	0.2	.	0.94	0.7658	0.7717	Clonal	A	.	.	.	Likely_pathogenic	TRUE	TRUE	TRUE
BoPT03	SETD2	p.Arg1625Cys	missense_variant	3	47144880	G	A	319	0.06	.	0.26	0.0000	0.1609	.	D	Driver	PASSENGER/OTHER	.	Likely_pathogenic	TRUE	TRUE	TRUE
BoPT03	TERT	c.-59C>T	upstream_gene_variant	5	1295228	G	A	185	0.38	.	1	0.9618	0.9262	Clonal	.	.	.	.	Likely_pathogenic	.	.	TRUE
BoPT05	DOT1L	p.Gly1471Glu	missense_variant	19	2226932	G	A	313	0.05	.	0.59	0.1061	0.3650	.	N	Passenger	PASSENGER/OTHER	.	Non-pathogenic	.	.	.
BoPT05	MED12	p.Leu39_Asn47del	inframe_deletion	X	70339236	CCTTGAATGTAAAACAAGGTTTCAATAA	C	914	0.08	.	0.89	0.6574	0.6997	Clonal	.	.	.	Deleterious	Likely_pathogenic	.	TRUE	TRUE
BoPT05	TERT	c.-59C>T	upstream_gene_variant	5	1295228	G	A	297	0.17	.	1	0.9383	0.8906	Clonal	.	.	.	.	Likely_pathogenic	.	.	TRUE
BoPT06	ASXL1	p.Glu1423Glu	synonymous_variant	20	31024784	G	A	381	0.03	.	0.09	0.0000	0.0436	.	.	.	.	.	Non-pathogenic	TRUE	TRUE	TRUE
BoPT06	IRS1	p.Ala874fs	frameshift_variant	2	227660834	GC	G	514	0.33	.	0.89	0.4058	0.7800	.	A	.	.	.	Non-pathogenic	.	.	.
BoPT06	MED12	p.Gly44Ala	missense_variant	X	70339254	G	C	703	0.38	.	1	0.9348	0.9113	Clonal	D	Passenger	PASSENGER/OTHER	.	Non-pathogenic	.	TRUE	TRUE
BoPT06	RARA	p.Leu398Val	missense_variant	17	38512281	C	G	352	0.29	.	0.79	0.0353	0.6661	.	D	Passenger	PASSENGER/OTHER	.	Non-pathogenic	.	.	TRUE
BoPT06	TRAF7	p.Asp357Glu	missense_variant	16	2223540	C	G	176	0.15	LOH	0.25	0.0000	0.1713	.	D	Passenger	PASSENGER/OTHER	.	Non-pathogenic	.	.	TRUE
BoPT07	MED12	p.Gly44Val	missense_variant	X	70339254	G	T	579	0.36	.	1	0.9705	0.9718	Clonal	D	Passenger	PASSENGER/OTHER	.	Non-pathogenic	.	TRUE	TRUE
BoPT07	RARA	p.Ser287Leu	missense_variant	17	38510606	C	T	600	0.31	.	1	0.9667	0.9647	Clonal	D	Passenger	PASSENGER/OTHER	.	Non-pathogenic	.	.	TRUE
BoPT07	TERT	c.-59C>T	upstream_gene_variant	5	1295228	G	A	261	0.26	.	1	0.9589	0.9087	Clonal	.	.	.	.	Likely_pathogenic	.	.	TRUE
BoPT08	MED12	p.Gly44Ser	missense_variant	X	70339253	G	A	250	0.33	.	1	0.8555	0.8891	Clonal	D	Passenger	PASSENGER/OTHER	.	Non-pathogenic	.	TRUE	TRUE
BoPT08	MET	p.Ala1342Ala	synonymous_variant	7	116435977	C	T	288	0.2	.	0.96	0.8067	0.7335	Clonal	.	.	.	.	Non-pathogenic	.	TRUE	TRUE
BoPT08	RARA	p.Thr233del	disruptive_inframe_deletion	17	38508646	TCCA	T	308	0.18	.	0.88	0.6624	0.6821	Clonal	D	.	.	Neutral	Likely_pathogenic	.	.	TRUE
BoPT08	TERT	c.-59C>T	upstream_gene_variant	5	1295228	G	A	77	0.22	.	1	0.7880	0.6153	Clonal	.	.	.	.	Likely_pathogenic	.	.	TRUE
BoPT09	JAK3	p.Leu635His	missense_variant	19	17946743	A	T	427	0.03	.	0.2	0.0000	0.1182	.	D	Driver	CANCER	.	Likely_pathogenic	.	.	TRUE
BoPT09	KDR	p.Ala775Val	missense_variant	4	55964913	G	A	241	0.09	.	0.46	0.0001	0.2985	.	D	Passenger	CANCER	.	Likely_pathogenic	.	.	TRUE
BoPT09	MED12	p.Gly44Val	missense_variant	X	70339254	G	T	426	0.19	.	1	0.8260	0.7879	Clonal	D	Passenger	PASSENGER/OTHER	.	Non-pathogenic	.	TRUE	TRUE
BoPT09	PTPN11	p.Arg527His	missense_variant	12	112926960	G	A	150	0.1	.	0.62	0.1395	0.3801	.	D	Passenger	PASSENGER/OTHER	.	Non-pathogenic	TRUE	TRUE	TRUE
MaPT01	EGFR	p.Gly63Arg	missense_variant	7	55210077	G	A	409	0.39	.	1	0.9299	0.8790	Clonal	D	Driver	CANCER	.	Likely_pathogenic	TRUE	TRUE	TRUE
MaPT01	TERT	c.-59C>T	upstream_gene_variant	5	1295228	G	A	217	0.41	.	1	0.9203	0.8533	Clonal	.	.	.	.	Likely_pathogenic	.	.	TRUE
MaPT02	CDH1	p.Glu745*	stop_gained	16	68862145	G	T	314	0.08	LOH	0.3	0.0000	0.2005	.	A	.	.	.	Likely_pathogenic	TRUE	TRUE	TRUE
MaPT02	ERBB3	p.Val104Leu	missense_variant	12	56478854	G	T	494	0.06	LOH	0.21	0.0000	0.1434	.	D	Passenger	PASSENGER/OTHER	.	Non-pathogenic	.	TRUE	.
MaPT02	FAT1	p.Ser377Ser	synonymous_variant	4	187629851	A	G	382	0.05	.	0.21	0.0000	0.1287	.	.	.	.	.	Non-pathogenic	.	TRUE	.
MaPT02	RB1	p.Cys706Phe	missense_variant	13	49037877	G	T	282	0.23	LOH	0.87	0.6234	0.6884	Clonal	D	Driver	CANCER	.	Likely_pathogenic	TRUE	TRUE	TRUE
MaPT02	RET	p.Ser406Gly	missense_variant	10	43604631	A	G	337	0.34	LOH	1	0.6382	0.9149	Clonal	N	Passenger	CANCER	.	Non-pathogenic	.	.	TRUE
MaPT02	RUNX1	p.Asp93Tyr	missense_variant	21	36259214	C	A	523	0.05	.	0.24	0.0000	0.1578	.	D	Passenger	CANCER	.	Likely_pathogenic	TRUE	TRUE	TRUE
MaPT02	TP53	p.Ser241Tyr	missense_variant	17	7577559	G	T	374	0.3	LOH	1	0.9422	0.8793	Clonal	D	Driver	CANCER	.	Likely_pathogenic	TRUE	TRUE	TRUE
MaPT03	MED12	p.Gly44Asp	missense_variant	X	70339254	G	A	714	0.36	.	1	0.7922	0.9779	Clonal	D	Passenger	PASSENGER/OTHER	.	Non-pathogenic	.	TRUE	TRUE
MaPT03	SETD2	p.Asp1616His	missense_variant	3	47144907	C	G	378	0.28	.	1	0.9263	0.9375	Clonal	D	Passenger	PASSENGER/OTHER	.	Non-pathogenic	TRUE	TRUE	TRUE
MaPT03	SETD2	p.Ser1777Phe	missense_variant	3	47127752	G	A	406	0.11	.	0.71	0.0746	0.5335	.	D	Passenger	PASSENGER/OTHER	.	Non-pathogenic	TRUE	TRUE	TRUE
MaPT03	SF3B1	p.Gly83Gly	synonymous_variant	2	198285804	T	C	385	0.09	.	0.55	0.0006	0.3952	.	.	.	.	.	Non-pathogenic	TRUE	TRUE	TRUE
MaPT03	TERT	c.-59C>T	upstream_gene_variant	5	1295228	G	A	181	0.28	.	1	0.9388	0.8784	Clonal	.	.	.	.	Likely_pathogenic	.	.	TRUE
MaPT04	ATRX	p.Gln929Glu	missense_variant	X	76937963	G	C	266	0.05	.	0.12	0.0000	0.0638	.	P	Passenger	PASSENGER/OTHER	.	Non-pathogenic	TRUE	.	TRUE
MaPT04	KMT2D	p.Trp2049*	stop_gained	12	49435737	C	T	345	0.39	.	1	0.9180	0.8698	Clonal	A	.	.	.	Likely_pathogenic	TRUE	TRUE	TRUE
MaPT04	NF1	.	splice_acceptor_variant&intron_variant	17	29508438	A	G	267	0.4	.	1	0.9217	0.8685	Clonal	D	.	.	.	Likely_pathogenic	TRUE	TRUE	TRUE
MaPT04	TSC2	p.Leu191Leu	synonymous_variant	16	2105494	C	T	259	0.35	.	0.93	0.7497	0.7730	Clonal	.	.	.	.	Non-pathogenic	.	.	TRUE
MaPT05	ATM	p.Asp1848del	inframe_deletion	11	108175444	CATG	C	342	0.36	.	1	0.9429	0.9486	Clonal	D	.	.	Deleterious	Likely_pathogenic	TRUE	TRUE	TRUE
MaPT05	EGFR	p.Glu84Val	missense_variant	7	55211008	A	T	220	0.2	.	1	0.8300	0.7271	Clonal	D	Passenger	CANCER	.	Likely_pathogenic	TRUE	TRUE	TRUE
MaPT06	ERBB2	p.Val777Leu	missense_variant	17	37881000	G	T	527	0.03	.	0.14	0.0000	0.0850	.	D	Driver	CANCER	.	Likely_pathogenic	.	TRUE	TRUE
MaPT06	RB1	p.Asp156fs	frameshift_variant	13	48919301	GA	G	244	0.31	LOH	0.97	0.8737	0.7770	Clonal	A	.	.	.	Likely_pathogenic	TRUE	TRUE	TRUE
MaPT06	TP53	p.Arg342*	stop_gained	17	7574003	G	A	389	0.41	LOH	1	0.2076	0.9381	Clonal	A	.	.	.	Likely_pathogenic	TRUE	TRUE	TRUE
MaPT08	NF1	p.Gln2492*	stop_gained	17	29679291	C	T	178	0.8	LOH	1	0.0000	0.9646	Clonal	A	.	.	.	Likely_pathogenic	TRUE	TRUE	TRUE
MaPT08	RB1	p.Asn849Ile	missense_variant	13	49050862	A	T	118	0.55	LOH	1	0.9384	0.8424	Clonal	D	Passenger	CANCER	.	Likely_pathogenic	TRUE	TRUE	TRUE
MaPT08	TP53	p.Arg342Pro	missense_variant	17	7574002	C	G	254	0.81	LOH	1	0.0000	0.9750	Clonal	N	Driver	CANCER	.	Likely_pathogenic	TRUE	TRUE	TRUE
MaPT10	EGFR	p.Leu62Arg	missense_variant	7	55210075	T	G	332	0.61	.	1	0.9864	0.9736	Clonal	D	Driver	PASSENGER/OTHER	.	Likely_pathogenic	TRUE	TRUE	TRUE
MaPT10	NRAS	p.Gln61Lys	missense_variant	1	115256530	G	T	162	0.41	.	0.96	0.8275	0.7683	Clonal	D	Driver	CANCER	.	Likely_pathogenic	TRUE	TRUE	TRUE
MaPT10	PDGFRB	p.Arg561Cys	missense_variant	5	149505134	G	A	208	0.4	.	0.95	0.8206	0.7833	Clonal	D	Passenger	CANCER	.	Likely_pathogenic	.	.	TRUE
MaPT10	PIK3CA	p.Glu453Gly	missense_variant	3	178928080	A	G	139	0.45	.	1	0.9020	0.8131	Clonal	D	Driver	CANCER	.	Likely_pathogenic	TRUE	TRUE	TRUE
MaPT10	PIK3CA	p.His1047Arg	missense_variant	3	178952085	A	G	174	0.38	.	0.88	0.6117	0.7091	Clonal	D	Driver	CANCER	.	Likely_pathogenic	TRUE	TRUE	TRUE
MaPT10	SETD2	p.Gln1794fs	frameshift_variant	3	47127702	G	GT	286	0.36	.	0.85	0.3383	0.7227	.	A	.	.	.	Likely_pathogenic	TRUE	TRUE	TRUE
MaPT10	TERT	c.-59C>T	upstream_gene_variant	5	1295228	G	A	184	0.52	.	1	0.8633	0.9049	Clonal	.	.	.	.	Likely_pathogenic	.	.	TRUE
MaPT12	AMER1	p.Asp537fs	frameshift_variant	X	63411556	GT	G	1122	0.11	LOH	0.42	0.0000	0.3480	.	A	.	.	.	Likely_pathogenic	.	.	TRUE
MaPT12	ATR	p.Leu2091Ser	missense_variant	3	142188975	A	G	300	0.13	.	0.48	0.0000	0.3493	.	D	Driver	CANCER	.	Likely_pathogenic	TRUE	.	.
MaPT12	TERT	c.-59C>T	upstream_gene_variant	5	1295228	G	A	279	0.19	.	0.92	0.6904	0.7064	Clonal	.	.	.	.	Likely_pathogenic	.	.	TRUE
MaPT12	TP53	p.Tyr220*	stop_gained	17	7578189	A	T	466	0.18	.	0.67	0.0002	0.5466	.	A	.	.	.	Likely_pathogenic	TRUE	TRUE	TRUE

Sample ID	Gene	Amino Acid Change	Effect	Chromosome	Pos	Ref	Alt	Tumor Depth	Tumor MAF	LOH	CCF	Probabilty Mutation is clonal	95% Confidence Interval (Low)	Clonality	Mutation Taster	CHASM	FATHMM	PROVEAN	Pathogenicity	Kandoth	Lawrence	Cancer Gene Census
MaPT13	BTK	p.Val173Ile	missense_variant	X	100617654	C	T	386	0.28	.	0.87	0.5272	0.7346	Clonal	N	Passenger	PASSENGER/OTHER	.	Non-pathogenic	.	.	.
MaPT13	MED12	p.Gly44Val	missense_variant	X	70339254	G	T	527	0.54	.	1	0.0001	0.9770	Clonal	D	Passenger	PASSENGER/OTHER	.	Non-pathogenic	.	TRUE	TRUE
MaPT13	PIK3CB	p.Leu385Leu	synonymous_variant	3	138433457	C	T	295	0.33	.	1	0.9057	0.8267	Clonal	.	.	.	.	Non-pathogenic	.	.	.
MaPT13	PRDM1	p.Arg112fs	frameshift_variant	6	106543527	G	GCACA	407	0.61	LOH	1	0.0006	0.9647	Clonal	A	.	.	.	Likely_pathogenic	.	TRUE	TRUE
MaPT13	SETD2	p.Arg2122fs	frameshift_variant	3	47098909	CG	C	443	0.33	.	1	0.9292	0.8640	Clonal	A	.	.	.	Likely_pathogenic	TRUE	TRUE	TRUE
MaPT13	SETD2	p.Ile270del	inframe_deletion	3	47165315	ATAT	A	462	0.3	.	0.92	0.7321	0.7852	Clonal	N	.	.	Neutral	Non-pathogenic	TRUE	TRUE	TRUE
MaPT13	TERT	c.-59C>T	upstream_gene_variant	5	1295228	G	A	166	0.41	.	1	0.8628	0.8756	Clonal	.	.	.	.	Likely_pathogenic	.	.	TRUE
AS3	ATRX	p.Gly2075Trp	NON_SYNONYMOUS_CODING	X	76829818	C	A	438	0.03	.	0.13	0.0000	0.0715	Subclonal	D	Passenger	PASSENGER/OTHER	.	Non-pathogenic	TRUE	.	TRUE
AS3	DICER1	p.Asp1709Asn	NON_SYNONYMOUS_CODING	14	95560464	C	T	81	0.5	.	1	0.8947	0.8883	Clonal	D	Passenger	CANCER	.	Likely pathogenic	.	.	TRUE
AS3	DICER1	p.Ser290*	STOP_GAINED	14	95592951	G	C	206	0.43	.	1	0.9731	0.9399	Clonal	A	Driver	CANCER	.	Likely pathogenic	.	.	TRUE
AS3	TP53	p.Arg342*	STOP_GAINED	17	7574003	G	A	98	0.86	.	1	0.1155	0.9526	Clonal	A	.	.		Likely pathogenic	TRUE	TRUE	TRUE
AS5	KMT2C	p.Gln755*	STOP_GAINED	7	151945256	G	A	92	0.21	LOH	0.67	0.2658	0.4397	Subclonal	N	.	.		Likely pathogenic	TRUE	TRUE	TRUE
AS5	MED12	p.Asp23Tyr	NON_SYNONYMOUS_CODING	X	70338671	G	T	272	0.06	.	0.31	0.0000	0.1879	Subclonal	D	Passenger	PASSENGER/OTHER		Non-pathogenic	.	TRUE	TRUE
AS5	MITF	p.Glu192*	STOP_GAINED	3	69987192	G	T	388	0.38	.	1	0.9856	0.9610	Clonal	A	.	.		Likely pathogenic	.	.	TRUE
AS5	PIK3CA	p.Asn1044Thr	NON_SYNONYMOUS_CODING	3	178952076	A	C	443	0.06	.	0.31	0.0000	0.2077	Subclonal	D	Driver	CANCER		Likely pathogenic	TRUE	TRUE	TRUE
AS6	DIS3	p.Asp479Glu	NON_SYNONYMOUS_CODING	13	73346363	G	C	265	0.39	.	1	0.8089	0.9380	Clonal	D	Passenger	PASSENGER/OTHER		Non-pathogenic	.	TRUE	.
AS8	AXIN1	p.Arg403Trp	NON_SYNONYMOUS_CODING	16	354351	G	A	55	0.45	.	1	0.0114	0.8490	Subclonal	D	Passenger	CANCER		Likely pathogenic	.	.	TRUE
BAS03	KDM5C	p.Ser1178fs	FRAME_SHIFT	X	53223817	GCCGTGCTC	G	1338	0.11	.	0.34	0.0000	0.2830	Subclonal	A	.	.		Likely pathogenic	TRUE	TRUE	TRUE
BAS03	FGFR2	p.Pro253Arg	NON_SYNONYMOUS_CODING	10	123279674	G	C	314	0.04	.	0.11	0.0000	0.0584	Subclonal	A	Passenger	CANCER		Likely pathogenic	TRUE	TRUE	TRUE
BAS03	BRCA2	p.Ala3205Pro	NON_SYNONYMOUS_CODING	13	32971146	G	C	190	0.03	.	0.1	0.0000	0.0423	Subclonal	N	Passenger	PASSENGER/OTHER		Non-pathogenic	TRUE	.	TRUE
BAS04	BBC3	p.Ala176Gly	NON_SYNONYMOUS_CODING	19	47729862	G	C	777	0.07	.	0.78	0.2689	0.5946	Subclonal	D		PASSENGER/OTHER		Non-pathogenic	.	.	.
BAS06	GRIN2A	p.Arg1309Trp	NON_SYNONYMOUS_CODING	16	9857476	G	A	225	0.43	.	0.88	0.4382	0.7441	Subclonal	D	Passenger	CANCER		Likely pathogenic	.	.	.
BAS06	GNAS	p.Arg844Ser	NON_SYNONYMOUS_CODING	20	57484420	C	A	272	0.39	.	0.79	0.0152	0.6748	Subclonal	D	Passenger	CANCER		Likely pathogenic	.	.	TRUE
BAS07	KEAP1	p.Ser104Arg	NON_SYNONYMOUS_CODING	19	10610400	T	G	794	0.23	.	1	0.8923	0.8539	Clonal	D	Passenger	PASSENGER/OTHER		Non-pathogenic	TRUE	TRUE	.
BAS09	PTCH1	p.Trp863fs	FRAME_SHIFT	9	98224253	C	CA	111	0.39	.	0.98	0.8143	0.7349	Clonal	A	.	.		Likely pathogenic	.	.	TRUE
BAS09	SMARCB1	p.Leu356fs	FRAME_SHIFT	22	24175835	ACT	A	89	0.61	.	0.93	0.8153	0.7592	Clonal	D	.	.		Likely pathogenic	.	TRUE	TRUE
BAS10	FLT4	p.Asn515Ser	NON_SYNONYMOUS_CODING	5	180050939	T	C	476	0.14	.	1	0.8611	0.7835	Clonal	D	Passenger	PASSENGER/OTHER		Non-pathogenic	.	.	.
BAS16	PIK3CG	p.Ala78Pro	NON_SYNONYMOUS_CODING	7	106508238	G	C	209	0.1	.	0.46	0.0001	0.2994	Subclonal	D	Passenger	CANCER		Likely pathogenic	TRUE	.	.
BAS16	PAK1	p.Leu465Phe	NON_SYNONYMOUS_CODING	11	77047151	G	A	114	0.32	.	1	0.7891	0.8195	Clonal	D	Passenger	PASSENGER/OTHER		Non-pathogenic	.	.	.
BAS16	DICER1	p.Asp1810Tyr	NON_SYNONYMOUS_CODING	14	95557639	C	A	123	0.61	.	1	0.0249	0.9424	Clonal	D	Passenger	CANCER		Likely pathogenic	.	.	TRUE
BAS18	KMT2C	p.Lys339Asn	NON_SYNONYMOUS_CODING	7	151962290	C	G	79	0.08	.	0.47	0.0992	0.2170	Subclonal	D	Passenger	PASSENGER/OTHER		Non-pathogenic	TRUE	TRUE	TRUE
BAS18	KMT2C	p.Arg284Gln	NON_SYNONYMOUS_CODING	7	151970951	C	T	46	0.13	.	0.81	0.5183	0.3484	Clonal	D	Passenger	PASSENGER/OTHER		Non-pathogenic	TRUE	TRUE	TRUE
BAS19	NSD1	p.Asn275fs	FRAME_SHIFT	5	176562926	AAATTT	A	700	0.05	.	0.11	0.0000	0.0749	Subclonal	A	.	.		Likely pathogenic	TRUE	TRUE	TRUE
BAS19	ABL1	p.Ser1063Pro	NON_SYNONYMOUS_CODING	9	133760807	T	C	825	0.07	.	0.01	0.0000	0.0545	Subclonal	D	Passenger	PASSENGER/OTHER		Non-pathogenic	.	.	TRUE
BAS19	FGFR2	p.Asn549Lys	NON_SYNONYMOUS_CODING	10	123258034	A	T	778	0.07	.	0.15	0.0000	0.1127	Subclonal	D	Driver	CANCER		Likely pathogenic	TRUE	TRUE	TRUE
BAS19	AXIN2	p.Ile157Thr	NON_SYNONYMOUS_CODING	17	63554269	A	G	831	0.06	.	0.12	0.0000	0.0902	Subclonal	D	Passenger	PASSENGER/OTHER		Non-pathogenic	TRUE	TRUE	.
BAS19	PIK3R1	p.Arg577fs	FRAME_SHIFT	5	67591136	AG	A	74	0.07	.	0.15	0.0000	0.0887	Subclonal	A	.	.		Likely pathogenic	TRUE	TRUE	TRUE
BAS19	PTEN	p.Ala151fs	FRAME_SHIFT	10	89692967	G	GC	65	0.05	.	0.11	0.0000	0.0504	Subclonal	A	.	.		Likely pathogenic	TRUE	TRUE	TRUE
BAS19	PTEN	p.Thr319fs	FRAME_SHIFT	10	89720803	T	TAA	121	0.05	.	0.11	0.0000	0.0607	Subclonal	A	.	.		Likely pathogenic	TRUE	TRUE	TRUE
BAS22	NKX3-1	p.Arg66Cys	NON_SYNONYMOUS_CODING	8	23540207	G	A	552	0.05	.	0.17	0.0000	0.1128	Subclonal	N	Passenger	PASSENGER/OTHER		Non-pathogenic	.	.	.
BAS27	SPOP	p.Arg121Gln	NON_SYNONYMOUS_CODING	17	47696461	C	T	481	0.07	.	0.78	0.3852	0.5512	Subclonal	D	.	PASSENGER/OTHER		Non-pathogenic	TRUE	TRUE	.
