# Supplementary material for: Genetic analysis of uterine adenosarcomas and phyllodes tumors of the breast
Source: Mol Oncol. 2017 May 16;11(8):913–26. doi: 10.1002/1878-0261.12049 (PMC5537914; doi:10.1002/1878-0261.12049)
Supplement: Supplementary file 8 — Table S3. Pathway analysis using gProfiler, MsigDB and DAVID in uterine adenosarcomas and phyllodes tumors of the breast included in this study. [file MOL2-11-913-s008.pdf]

**Supplementary Table S3: Pathway analysis using gProfiler, MsigDB and DAVID in uterine adenosarcomas and phyllodes tumors of the breast.**

| Tumor type | Method | Database/ gene set ID | Description/ Term | Number of genes in gene set | Number of genes in overlap | p-value |
|---|---|---|---|---|---|---|
| Adenosarcoma | gProfiler | GO:0014070 | Response to organic cyclic compound | 80 | 29 | 0.0343 |
| | | GO:0071407 | Cellular response to organic cyclic compound | 52 | 22 | 0.0271 |
| | MsigDB | BIOCARTA_CTCF_PATHWAY | CTCF: First Multivalent Nuclear Factor | 23 | 8 | 5.62E-18 |
| | | KEGG_P53_SIGNALING_PATHWAY | p53 signaling pathway | 69 | 10 | 5.81E-18 |
| | | PID_RB_1PATHWAY | Regulation of retinoblastoma protein | 65 | 11 | 1.88E-20 |
| | DAVID | KEGG_PATHWAY_hsa04115 | p53 signaling pathway | 46 | 9 | 0.021739996 |
| | | KEGG_PATHWAY_hsa05220 | Chronic myeloid leukemia | 46 | 13 | 0.025718424 |
| | | **KEGG_PATHWAY_hsa04310** | **Wnt signaling pathway** | **46** | **8** | **0.035206157** |
| | | KEGG_PATHWAY_hsa05217 | Basal cell carcinoma | 46 | 5 | 0.036249714 |
| | | **PANTHER_PATHWAY_P00057** | **Wnt signaling pathway** | **30** | **9** | **0.045112014** |
| All phyllodes tumors | gProfiler | GO:1902105 | Regulation of leukocyte differentiation | 29 | 11 | 0.0437 |
| | | GO:0045619 | Regulation of lymphocyte differentiation | 15 | 8 | 0.0282 |
| | mSigDB | DING_LUNG_CANCER_MUTATED_SIGNI | The lung adenocarcinoma TSP (tumor sequencing project) genes that were found significantly mutated by at least one method. | 26 | 8 | 3.70E-19 |
| | | TCGA_GLIOBLASTOMA_MUTATED | Genes significantly mutated in 91 glioblastoma samples. | 8 | 6 | 1.12E-17 |
| | | PID_TCPTP_PATHWAY | Signaling events mediated by TCPTP | 43 | 8 | 3.39E-17 |
| | | KEGG_NON_SMALL_CELL_LUNG_CANC | Non-small cell lung cancer | 54 | 8 | 2.42E-16 |
| | | PID_SHP2_PATHWAY | SHP2 signaling | 58 | 8 | 4.44E-16 |
| | DAVID | REACTOME_PATHWAY R-HSA-5673001 | RAF/MAP kinase cascade | 36 | 9 | 7.15E-03 |
| | | REACTOME_PATHWAY R-HSA-2219530 | Constitutive Signaling by aberrant PI3K in Cancer | 36 | 8 | 1.66E-02 |
| | | REACTOME_PATHWAY R-HSA-3214841 | PKMTs methylate histone lysines | 36 | 5 | 1.80E-02 |
| | | KEGG_PATHWAY_hsa05166 | HTLV-I infection | 34 | 12 | 2.79E-02 |
| | | KEGG_PATHWAY_hsa00310 | Lysine degradation | 34 | 4 | 2.81E-02 |
| | | REACTOME_PATHWAY R-HSA-1963642 | PI3K events in ERBB2 signaling | 36 | 4 | 2.96E-02 |
| | | BIOCARTA_h_g1Pathway | Cell Cycle: G1/S Check Point | 22 | 6 | 3.88E-02 |
| | | REACTOME_PATHWAY_R-HSA-1250196 | SHC1 events in ERBB2 signaling | 36 | 4 | 4.72E-02 |
| Benign phyllodes tumors | MsigDB | **PID_BETA_CATENIN_NUC_PATHWAY** | **Regulation of nuclear beta catenin signaling and target gene transcription** | **80** | **3** | **0.000000177** |
| | DAVID | REACTOME_PATHWAY_R-HSA-3214841 | PKMTs methylate histone lysines | 6 | 3 | 6.02E-03 |
| | | **REACTOME_PATHWAY_R-HSA-201722** | **Formation of the beta-catenin-TCF transactiovating complex** | **6** | **3** | **6.80E-03** |
| Borderline phyllodes tumors | MsigDB | BIOCARTA_CELLCYCLE_PATHWAY | Cyclins and Cell Cycle Regulation | 23 | 3 | 3.12E-08 |
| | | BIOCARTA_G1_PATHWAY | Cell Cycle: G1/S Check Point | 28 | 3 | 5.77E-08 |
| | | PID_AVB3_INTEGRIN_PATHWAY | Integrins in angiogenesis | 75 | 3 | 1.18E-06 |
| | | **PID_BETA_CATENIN_NUC_PATHWAY** | **Regulation of nuclear beta catenin signaling and target gene transcription** | **80** | **3** | **1.43E-06** |
| | | PID_IL2_1PATHWAY | IL2-mediated signaling events | 55 | 3 | 4.60E-07 |
| | | PID_IL2_PI3K_PATHWAY | IL2 signaling events mediated by PI3K | 34 | 3 | 1.05E-07 |
| | | PID_SHP2_PATHWAY | SHP2 signaling | 58 | 4 | 1.62E-09 |
| | | REACTOME_G1_PHASE | Genes involved in G1 Phase | 38 | 3 | 1.48E-07 |
| Malignant phyllodes tumors | gProfiler | GO:0048869 | Cellular developmental process | 205 | 24 | 0.0242 |
| | MsigDB | TCGA_GLIOBLASTOMA_MUTATED | Genes significantly mutated in 91 glioblastoma samples. | 8 | 6 | 6.33E-19 |
| | | KEGG_NON_SMALL_CELL_LUNG_CANC | Non-small cell lung cancer | 54 | 7 | 1.80E-15 |
| | | DING_LUNG_CANCER_MUTATED_SIGNI | The lung adenocarcinoma TSP (tumor sequencing project) genes that were found significantly mutated by at least one method. | 26 | 6 | 5.17E-15 |
| | | KEGG_BLADDER_CANCER | Bladder cancer | 42 | 6 | 1.17E-13 |
| | DAVID | REACTOME_PATHWAY_R-HSA-1963642 | PI3K events in ERBB2 signaling | 21 | 4 | 0.005880118 |
| | | REACTOME_PATHWAY_R-HSA-1250196 | SHC1 events in ERBB2 signaling | 21 | 4 | 0.009804934 |
| | | KEGG_PATHWAY_hsa05219 | Bladder cancer | 20 | 6 | 0.016894989 |
| | | BIOCARTA_h_telPathway | Telomeres, Telomerase, Cellular Aging, and Immortality | 13 | 4 | 0.020804977 |
| | | KEGG_PATHWAY_hsa05230 | Central carbon metabolism in cancer | 20 | 7 | 0.023743726 |
| | | REACTOME_PATHWAY_R-HSA-5673001 | RAF/MAP kinase cascade | 21 | 6 | 0.025619796 |
| | | KEGG_PATHWAY_hsa04020 | Calcium signaling pathway | 20 | 4 | 0.028161439 |
| | | BIOCARTA_h_plk3Pathway | Regulation of cell cycle progression by Plk3 | 13 | 3 | 0.045068294 |
| | | BIOCARTA_h_rbPathway | RB Tumor Suppressor/Checkpoint Signaling in response to DNA damage | 13 | 3 | 0.045068294 |
| | | KEGG_PATHWAY_hsa05166 | HTLV-I infection | 20 | 8 | 0.047652915 |

**Note: Wnt signaling and nuclear beta-catenin pathways are highlighted in bold.**
